# A simplified counter-selection recombineering protocol for creating fluorescent protein reporter constructs directly from *C. elegans* fosmid genomic clones

**DOI:** 10.1186/1472-6750-13-1

**Published:** 2013-01-03

**Authors:** Nisha Hirani, Marcel Westenberg, Minaxi S Gami, Paul Davis, Ian A Hope, Colin T Dolphin

**Affiliations:** 1 Institute of Pharmaceutical Science, King’s College London, Franklin-Wilkins Building, 150 Stamford Street, London, SE1 9NH, UK; 2 EMBL-EBI, Wellcome Trust Genome Campus, Hinxton, Cambridge, CD10 1SD, UK; 3 Institute of Integrative and Comparative Biology, Faculty of Biological Sciences, University of Leeds, Leeds, LS2 9JT, UK; 4 Present address: Plant Protection Service (NPPO), National Reference Centre, Department of Molecular Biology, P.O. Box 9102, Wageningen, 6700HC, The Netherlands

**Keywords:** *C*. *elegans*, Recombineering, Fosmid, Fluorescent protein, Deoxyribose-phosphate aldolase, Peroxiredoxin, Metallocarboxypeptidase

## Abstract

**Background:**

Recombineering is a genetic engineering tool that enables facile modification of large episomal clones, e.g. BACs, fosmids. We have previously adapted this technology to generate, directly from fosmid-based genomic clones, fusion gene reporter constructs designed to investigate gene expression patterns in *C*. *elegans*. In our adaptation a *rpsL-tet(A)* positive/negative-selection cassette (RT-cassette) is first inserted and then, under negative selection, seamlessly replaced with the desired sequence. We report here on the generation and application of a resource comprising two sets of constructs designed to facilitate this particular recombineering approach.

**Results:**

Two complementary sets of constructs were generated. The first contains different fluorescent protein reporter coding sequences and derivatives while the second set of constructs, based in the copy-number inducible vector pCC1Fos, provide a resource designed to simplify RT-cassette-based recombineering. These latter constructs are used in pairs the first member of which provides a template for PCR-amplification of an RT-cassette while the second provides, as an excised restriction fragment, the desired fluorescent protein reporter sequence. As the RT-cassette is flanked by approximately 200 bp from the ends of the reporter sequence the subsequent negative selection replacement step is highly efficient. Furthermore, use of a restriction fragment minimizes artefacts negating the need for final clone sequencing. Utilizing this resource we generated single-, double- and triple-tagged fosmid-based reporters to investigate expression patterns of three *C*. *elegans* genes located on a single genomic clone.

**Conclusions:**

We describe the generation and application of a resource designed to facilitate counter-selection recombineering of fosmid-based *C*. *elegans* genomic clones. By choosing the appropriate pair of ‘insertion’ and ‘replacement’ constructs recombineered products, devoid of artefacts, are generated at high efficiency. Gene expression patterns for three genes located on the same genomic clone were investigated via a set of fosmid-based reporter constructs generated with the modified protocol.

## Background

Since its introduction as a model metazoan animal [1] the experimental convenience, simple anatomy, which includes most differentiated tissues including a nervous system, and, in particular, genetic tractability has made the nematode *Caenorhabditis elegans* a popular research platform for many biologists and geneticists [2]. Sequence analysis of the *C. elegans* genome, completed in 1998 [3], has revealed the presence of approximately twenty thousand protein-coding genes. Although many of these have human homologues many are yet to have a function assigned. A number of complementary methodological approaches are available to functionally analyze *C. elegans* genes amongst which the determination of the spatial-temporal pattern of gene expression is particularly informative. While such patterns can be investigated at the level of transcription or translation, *via* approaches such as, respectively, *in situ* hybridization or immunohistochemistry, it is more common to employ reporter technology and analyze, in transgenic animals, the expression pattern of the surrogate reporter.

In its simplest guise, reporter technology involves cloning either the known promoter region or, if this sequence has not been precisely defined, as is often the case, then all or part of the 5^′^ intergenic sequence upstream of a reporter gene, such as *gfp*, in an appropriate base plasmid (e.g. reference [4]) to generate a so-called transcriptional reporter. Because the cloned sequence may not contain all necessary regulatory elements resulting expression patterns should be interpreted cautiously as they may not fully reflect that of the gene under study. If, on the other hand, a more complex translational reporter, in which the reporter is fused in-frame to the protein-coding sequence of the gene of interest, is constructed then, in addition to deriving information about promoter activity, potentially other related aspects of gene expression, such as the sub-cellular localization of the corresponding gene product, can also be derived. In order to include distantly located regulatory elements, and thus generate a construct more likely to recapitulate the expression pattern of the endogenous gene, such a reporter would, ideally, contain not only the genomic locus under study but also significant stretches of the 5^′^ and 3^′^ flanking sequence. Because *C. elegans* genes are relatively compact (5 kb on average [3]) and the fosmid-based genomic clone insert is typically 35–40 kb, a translational reporter based upon such a fosmid would, when the locus under study is located within the central region of the insert, provide that broad genomic DNA environment. Fosmid-based *C. elegans* genomic clones are thus ideal foundations for translational reporter construction [5].

To facilitate the generation of such reporter constructs from *C. elegans* fosmid clones we [5,6], and others [7-9], have developed tools and techniques designed to leverage the power of recombineering. Recombineering is a homologous recombination (HR)-based genetic engineering system mediated by transient expression of *λ*-encoded recombinases within an *E*. *coli* host (reviewed in [10]). We reported previously [5] on the use of a *rpsL-tet(A)* positive/negative-selection cassette (RT-cassette) [11] in a two-step counter-selection recombineering approach that enables the seamless creation of fosmid-based reporters devoid of undesired, extraneous sequence changes. Although some groups [8,9] have reported poor recombineering efficiencies using the RT-cassette as a selectable marker others [12,13] have, like us, employed it successfully to generate highly informative reporter fusions.

Although the various strategies and associated resources provide *C. elegans* researchers with a range of powerful recombineering tools the methodology is perhaps not as commonly practiced as it might be. To both further encourage its uptake and to facilitate an ongoing project designed to interrogate putative internal promoter elements within operons we generated two sets of constructs designed to provide tools to simplify the RT-cassette-based counter-selection recombineering approach employed in our laboratories. The first set contained coding sequences (CDSs) encoding potentially useful fluorescent protein (FP) reporters intended to expand the FP palette currently in common use by *C. elegans* researchers. In order to maximise the utility of the final resource, *N*- and *C*-terminal TAP- (tandem affinity purification) tag [14,15] sequences were also included. The FP-encoding sequences were both extracted from the previously described vectors pPD136.61, pPD136.64 [4] and pAA64 [16] that encode, respectively, CFP, YFP and the red-shifted mCherry [17], and synthesized *de novo* to encode the less frequently used mCerulean [18], mTFP1 (Teal FP) [19] and mCitrine [20]. A set of these inserts was subsequently introduced into the single-copy fosmid vector pCC1Fos generating the second set of constructs that provide the tools to faciliate RT-cassette-based recombineering. The resulting method requires only a single PCR, to generate the initial RT-cassette, and generates final products of high fidelity. We report here on the generation of these constructs and illustrate their utility by building reporter fusion constructs designed to examine the expression patterns of three *C. elegans* genes. These genes were all located on a single fosmid and a series of reporter constructs were built, *via* iterative rounds of counter-selection recombineering, in which one, two or all three genes were tagged with a different FP reporter. Fluorescence microscopy of lines transgenic for a subset of these constructs revealed expression patterns that either confirmed those previously reported or, in the case of one gene, indicated an unexpected aggregation of the fusion gene product. Unexpectedly, and somewhat disappointingly, mCerulean, mCitrine and mCherry, each encoded by a codon-optimized CDS, exhibited either rather fast bleaching or were not as bright as had been envisaged particularly when expressed as fusion proteins. As these characteristics make their corresponding constructs less useful than hoped only those constructs that provided useful recombineering tools are being made available *via* the plasmid depository Addgene. By selecting constructs from this resource appropriate to their aims *C. elegans* researchers will be able to recombineer fosmid-based reporter constructs in a simplified, streamlined manner.

## Results and discussion

Approximately 15% of all *C. elegans* genes are clustered together into polycistronic transcriptional units termed operons [21]. We have begun to investigate the extent that downstream operon genes may be transcriptionally regulated *via* internal *cis*-acting sequence elements (Hirani et al., unpublished). Operons containing such internal promoter elements, so-called hybrid operons, have been identified using transcriptional reporters designed to interrogate, in isolation, the intergenic regions between neighbouring operon genes [22]. In contrast, we are employing fosmid-based reporters to enable us to both manipulate precisely such putative internal regulatory elements and, at the same time, tag multiple operon genes with different FPs, without, in either case, otherwise disturbing the gross operon structure. To facilitate the recombineering of these reporter constructs we designed and built a resource consisting of two complementary sets of plasmids. The first of these comprises a modular set of FP CDSs including versions augmented by addition of *N-* and *C-*terminal TAP-tags or a nuclear-localization signal. The rationale for this set of plasmids was our initial aim to tag, with different FP reporters, up to four genes within any one operon. Spectral discrimination between each FP would be achieved by a combination of careful choice of FP with associated filter set and combining this, if necessary, with post-acquisition spectral unmixing. However, as discussed below, ultimately only three different FPs were combined within a single fosmid-based reporter enabling us to discriminate readily between their respective spectral signals simply using appropriate filter sets. The second set of plasmids, built using inserts from the first, was designed to both simplify and speed up the recombineering procedure and generate high fidelity FP fusion gene reporter products.

### Sequences encoding fluorescent protein reporters

To identify FPs with sufficient spectral separation to allow multiple tagging and, at the same time, minimize the requirement for computational separation of the resulting fluorescent signals we considered the relative biophysical and spectral properties of the FPs available [23,24]. Monomeric FPs with reported high photostability and brightness were selected from the cyan, green, yellow and red regions of the visible light spectrum and led to the choice of the cyan mCerulean [18], the cyan/green mTFP1 [19], the yellow mCitrine [20] and the red mCherry [17]. Although DNA sequences encoding each of the FPs were available we decided to design, and have commercially synthesized, novel CDSs as this would not only permit codon optimization but also, by careful choice and placement of restriction enzyme (RE) sites, enable us to simplify all envisaged downstream sub-cloning events. In addition, because introns have been demonstrated to generally increase levels of heterologous gene expression [25], and are routinely inserted into sequences encoding reporter proteins in *C. elegans*, we also designed and included silent, blunt-cutting internal RE sites to enable facile insertion of up to two artificial introns per CDS (Additional file 1: Figure S1 and Additional file 1: Table S1).

We expanded the overall utility of this first set of constructs by including additional sequences encoding *N*- and *C*-terminal TAP-tags (pHN001, pNH002) to enable combined gene expression analysis and TAP-based protein purification [14,15]. We chose, as the terminal epitope, the S-Tag sequence due to its strong retention on S-protein resins [26] followed by, as the protease site, the recognition sequence for human rhinovirus 3C protease. For the internal tag we decided upon StrepTag II [27] because of its highly specific interaction with Strep-Tactin resins and the ability to subsequently elute bound protein(s) under gentle conditions. Although not yet experimentally tested these TAP-tags can be excised from constructs available from Addgene (Table 1). The modular design of the synthesized sequences and their provision in a vector, pGOv5, stripped of common restriction sites enabled us to build quickly a range of sub-clones encompassing the more useful potential module combinations (Additional file 1: Table S1).

**Table 1:** pGOv5-based constructs^a^

**Construct**	**Insert**	**Intron 1^e^**	**Intron 2**
pNH001	[(G_4_S)_3_]::C-TAP-tag::2xNLS ^b^	-	-
pNH002	N-TAP-tag::[(G_4_S)_3_]::mTFP1 ^c^	-	-
pNH009	N-TAP-tag::mTFP1	-	-
pNH013	mTFP1 ^d^	-	-
pNH026	mTFP1::[(G_4_S)_3_]::C-TAP-tag	-	-
pNH030	mTFP1::[(G_4_S)_3_]::2xNLS	-	-
pNH078	mTFP1[1I] ^d^	B	-
pNH082	mTFP1[2I] ^d^	B	C

^a^ All constructs are in *E. coli* DH5α and are available from Addgene ^b^ lacks intact open-reading frame; ^c^ mTFP1 sequence lacks N-terminal Met-Val and C-terminal Lys residues present in native sequence; ^d^ N-terminal Met-Val residues and C-terminal Lys residue, present in native coding sequence, replaced, respectively, with Met-Ala-Ala and Val-Ser-Ala; ^e^ see Additional file 1: Table S2.

Prior to their incorporation into fosmid-based reporters the spectral and biophysical properties of the FPs were briefly investigated *via in vitro* and *in vivo* approaches. First, each FP was expressed *in vitro* from a DNA template and the resulting protein immunocaptured to agarose beads *via* an appropriate anti-FP antibody. Fluorescence microscopy revealed discrete, concentrated fluorescence of the intended colour (Additional file 1: Figure S5) confirming that each FP had been successfully generated from its respective CDS and each exhibited the expected spectral profile. Second, we observed fluorescence expression patterns in transgenic *C. elegans* strains transformed with constructs built from the *myo*-3^PROM^-containing vector pPD95.86 [4] that encoded either an unembellished FP, with a contiguous or two intron-containing (2I) CDS, or with a C-terminal nuclear localization signal (NLS). Control strains were also generated by transformation with DNA of equivalent pPD95.86-based sub-clones that contained CDSs encoding CFP, YFP and mCherry excised, respectively, from pPD136.61, pPD136.64 [4] and pAA64 [16]. To distinguish these FPs from those encoded by CDSs designed as part of this work the prior versions of CFP and YFP are prefixed with F (F-CFP, F-YFP) and mCherry with Mc (Mc-mCherry). All strains revealed the expected body muscle expression pattern (Additional file 1: Figure S5) with clear nuclear localization for the FPs equipped with a NLS (data not shown). Although these results indicated that the FPs possessed the anticipated spectral properties, both *in vitro* and *in vivo*, two additional observations were made. First, rapid photobleaching was exhibited by mCerulean, particularly when expressed *in vivo* within transgenic worms, using the same illumination intensity and exposure time parameters employed to visualize the other FPs including F-CFP. As the FP had effectively completely bleached in the time it took to focus on the fine detail of a specimen it was concluded that mCerulean would be impractical as a FP partner in multiple *in vivo* gene tagging strategies and that the three FP combination mTFP1/mCitrine/mCherry would be used in future constructs. The reasons for the rapid *in vivo* fluorescence decay of mCerulean, especially in comparison to that observed with the ancestral enhanced CFP (ECFP) (visualized here as F-CFP), is not clear as the amino acid sequence is the same as that of the original mCerulean [18]. Interestingly, recent crystallographic data [28] has suggested that the additional Y145A change present in mCerulean might exacerbate the likelihood of fluorescence quenching in comparison to CFP. Whatever the underlying cause(s), further improved versions of cyan-shifted FPs have been developed, e.g. mCerulean3 [29] and mTurquoise2 [28], which would be less likely to suffer from such fluorescence instability.

The second observation was the apparent lack of improvement, in terms of relative brightness, exhibited by mCitrine and mCherry when compared, respectively, to F-YFP and Mc-mCherry despite the codon optimization undertaken specifically to maximize expression *in vivo*. Both latter FPs are encoded by CDSs containing three short, equally spaced artificial introns. As expected, the introduction of two similar sized artificial introns, albeit not spaced equally, into the CDSs encoding mCitrine and mCherry improved, as judged by eye, protein expression levels in transgenic worms over the equivalent non-intron-containing CDSs (data not shown). However, longer exposure times were still required to achieve fluorescence emissions equivalent to worms transformed with corresponding F-YFP- and Mc-mCherry-encoding constructs. In contrast, mTFP1 proved to be consistently bright and photostable requiring only rather short (2–5 msec) exposure times when encoded by the two intron-containing CDS. Many factors are involved in determining the overall brightness of the FP reporter including the efficiency of transcription/translation of the encoding sequence as well as the intrinsic spectral properties of the resulting fluorophore. With respect to the latter, we expected mCitrine, especially when encoded by the two-intron-containing CDS, to be at least as bright as F-YFP, when illuminated under equivalent exposure conditions, as this FP is considered superior to standard YFP in both brightness and stability [23,24]. Although strains transformed with *myo*-3^PROM^-driven Mc-mCherry reproducibly exhibited brighter images than equivalent strains transformed with mCherry the two FPs had identical amino acid sequences and differed only by being encoded by different, “codon-optimized” CDSs that were globally 77% identical (data not shown). The differences in brightness observed between the respective yellow and red FP pairs are more likely due to differences in the efficiency with which each CDS is transcribed and/or the resulting mRNA translated into protein resulting in reduced FP abundance than any significant differences between the spectral properties of the fluors. Although we attempted to ensure, by designing sequences from scratch, that the CDSs encoding mCitrine and mCherry would promote efficient transcription and translation it is clear that simple assumptions concerning, for example, adjusting codon choice to reflect usage in highly expressed endogenous genes, does not necessarily guarantee optimized protein expression [30].

### pCC1Fos-based constructs

The second set of plasmids, built with inserts derived from the first, was designed to enable the recombineering procedure to be simplified, take less time and generate final FP fusion gene reporter constructs of high fidelity. As a subset of the plasmids were to contain the RT-cassette which, we had found, confers instability when present in a standard high-copy number vector (unpublished observations), it was decided to base them all in pCC1Fos. As well as maintaining clones as single copies for stable, routine propagation pCC1Fos-based constructs can, when hosted in a suitable strain such as EPI300 or MW005 [6], also be transiently induced to approximately 50 copies per bacterial cell thus improving DNA isolation yield and purity. These plasmids were designed as pairs the first member of which provides the template for PCR-amplification of the RT-cassette, used in the first, positive selection recombineering step, while the second provides the desired replacement sequence, excised as a restriction fragment, for use in the subsequent counter selection step. Generation of this pCC1Fos-based construct set greatly simplifies the RT-cassette-mediated counter-selection recombineering protocol as only one PCR needs to be performed and a restriction fragment is used to replace the inserted RT-cassette in the second recombineering step. The first step involves PCR-amplification of an RT-cassette from a template sequence excised by restriction enzyme digestion from the appropriate construct. The product is flanked by approximately 200 bp, derived from the template, matching the 5^′^ and 3^′^ ends of the chosen FP CDS and 50 bp terminal homology arms, derived from the primers, designed to direct insertion into the chosen site within the target gene. Replacement of the inserted RT-cassette is achieved by recombineeering transformation with a *Not*I restriction fragment, excised from a second plasmid, containing the desired FP CDS. The inclusion of the extended regions of flanking FP CDS homology with the RT-cassette ensures this negative selection replacement step is highly efficient with far fewer false positives, e.g. from when the RT-cassette is deleted rather than being replaced. In our hands, for clones selected under negative selection with chloramphenicol and streptomycin, successful replacement of the RT-cassette with the desired sequence is essentially 100% (data not shown). This improvement in HR brings a number of additional methodological benefits including smaller amounts of linear replacement DNA (50 ng) being required and, as far fewer bacterial cells need be to electroporated, reduced cell culture volumes and shorter incubation times. Furthermore, use of a restriction fragment ensures final recombineered products are essentially free of sequence artefacts introduced by PCR requiring only RE analysis to confirm construction fidelity. Lists of paired constructs designed to provide template for PCR-amplification of the RT-cassette donor and the subsequent replacement fragment are provided in Table 2 and Additional file 1: Table S6. Additional methodological details are provided in the supplemental data.

**Table 2:** Resources for simplified counter-selection recombineering

			**RT-cassette-containing construct^b^**		**Replacement construct^c^**		
**Desired insertion sequence^a^**	**RT**	**kb^d^**	**Fwd (5^′^-3^′^)^e^**	**Rev (5^′^-3^′^)^e^**	**pCC1Fos-based**	**pGOv5-based**	**kb^f^**
RT-cassette	pNH034	2.0	GCTGTCGAGATATGACGGTGTTCA	TCTTGGAGTGGTGAATCCGTTAGC	-	-	
F-CFP	pNH050	2.4	ATGAGTAAAGGAGAAGAACTTTTC	[*]TTTGTATAGTTCATCCATGCCATG	pNH039	n/a	0.9
F-GFP	pNH051	2.4	ATGAGTAAAGGAGAAGAACTTTTC	[*]TTTGTATAGTTCATCCATGCCATG	pNH040	n/a	0.9
F-YFP	pNH052	2.4	ATGAGTAAAGGAGAAGAACTTTTC	[*]TTTGTATAGTTCATCCATGCCATG	pNH041	n/a	0.9
Mc-mCherry	pNH053	2.4	ATGGTCTCAAAGGGTGAAGAAGAT	[*]GGATCCACTAGTCTTATACAATTC	pNH042	n/a	0.9
mTFP1	pNH054	2.4	ATGGCCGCCTCAAAAGGAGAAGAA	[*]AGCGCTTACGTAGAGCTCGTCCAT	pNH043	pNH013	0.7
N-TAP-tag::[(G_4_S)_3_]::mTFP1	pNH066	2.6	ATGGTTAAAGAAACAGCAGCAGCG	[*]AGCGCTTACGTAGAGCTCGTCCAT	pNH058	pNH002	0.9
mTFP1::[(G_4_S)_3_]::C-TAP-tag	pNH094	2.3	ATGGCCGCCTCAAAAGGAGAAGAA	[*]AGCCCATGAGTCCATATGCTGTCT	pNH090	pNH026	0.9
mTFP1::[(G_4_S)_3_]::2xNLS	pNH070	2.6	ATGGCCGCCTCAAAAGGAGAAGAA	[*]AGCGCTAACTTTTCGCTTCTTCTT	n/a	pNH030	0.8
mTFP1[B]	pNH054	2.4	ATGGCCGCCTCAAAAGGAGAAGAA	[*]AGCGCTTACGTAGAGCTCGTCCAT	n/a	pNH078	0.8
mTFP1[BC]	pNH054	2.4	ATGGCCGCCTCAAAAGGAGAAGAA	[*]AGCGCTTACGTAGAGCTCGTCCAT	n/a	pNH082	0.8

^a^ sequence to be seamlessly inserted into target; ^b^ the counter-selection RT-cassette should be PCR-amplified from this construct using, as template, the RT-cassette-containing *Not*I-*Not*I fragment; ^c^ the desired replacement cassette can be excised, as a *Not*I-*Not*I fragment, from either the pCC1Fos- (if available) or parental pGOv5-based (Table 1) constructs; ^d^ size of the isolated *Not*I-fragment (or *Nco*I-*Nco*I fragment for pNH034) to be used as PCR template; ^e^ suggested 3^′^ sequences of ‘recombineering’ ODNs designed to PCR-amplify FP-flanked, RT-cassette-containing sequence. Reverse ODN sequence lacks a 5^′^ stop triplet [*]; ^f^ size of the isolated *Not*I-fragment to be used to replace the inserted FP-flanked, RT-cassette-containing sequence. All constructs are available from Addgene and are in *E. coli* EPI300 apart from those built in pGOv5 which are in *E. coli* DH5α.

### Gene expression analysis

**Figure 1:**
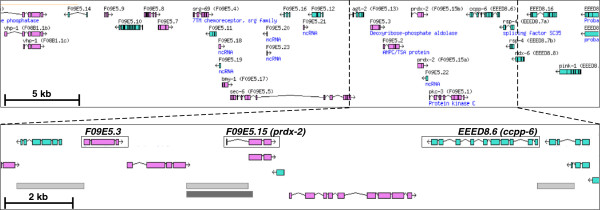
**Genomic location of fosmid WRM069dD11 insert.** Upper panel: genes on the forward (purple exons) and reverse (green exons) strands of the insert from fosmid genomic clone WRM069dD11. Lower panel: An expanded region of the insert demonstrating the relative locations of the genes (boxed) *F09E5.3*, *F09E5.15* (*prdx-2*) and *EEED8.6* (*ccpp-6*) for which published, independent expression data was available. Locations of the genomic regions used to drive *gfp* expression in the transcriptional reporters described by McKay *et al*. (2003) ref [32] and Hunt-Newbury *et al*. (2007) ref [33] (light grey boxes) for *F09E5.3*, *F09E5.15* and *EEED8.6*, strains BC14910, BC13145 and BC11803, respectively, and Isermann *et al*. (2004) ref [35] (dark grey box) for *F09E5.15* are indicated (lower panel). Figure derived from WormBase.

**Figure 2:**
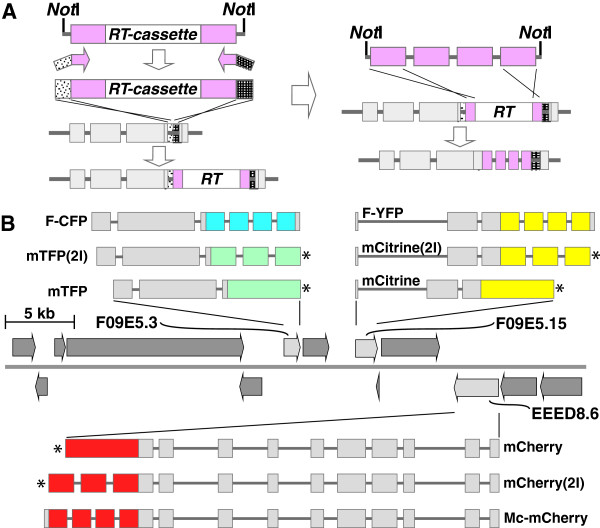
**Counter-selection recombineering-mediated generation of fosmid-based fluorescent protein-fusion reporter constructs.** Panel **A**: schematic representation of the modified counter-selection protocol. A *rps*L*-tet*A(C) (RT) counter-selection cassette, PCR-amplified from a *Not*I fragment excised from the appropriate pCC1Fos-based construct, flanked by approx. 200 bp from the 5^′^ and 3^′^ ends of the fluorescent protein reporter to be inserted (purple boxes) and terminal 50 bp homology arms (stippled boxes), is recombineered into the insertion site within the target gene *via* tetracycline selection of the positive marker (*tet*A(C)). In the subsequent replacement step a *Not*I fragment, containing the fluorescent protein coding sequence, replaces the inserted RT-cassette, *via* streptomycin selection conferred due to loss of the negative marker (*rps*L), generating an in-frame *gene::fp* fusion. Panel **B**: a schematic representation, within the centre of the panel and drawn approximately to scale, illustrating sizes and orientations of the genes located on the insert of the fosmid clone WRM069dD11. The genes *F09E5.3*, *F09E5.13* and *EEED8.6* (light grey solid arrows) were tagged, respectively, with one of either F-CFP, mTFP1(2I) or mTFP1, F-YFP, mCitrine(2I) or mCitrine, or Mc-mCherry, mCherry(2I) or mCherry. Reporter gene fusions marked with an asterisk lack the 5 terminal codons of the target gene.

**Figure 3:**
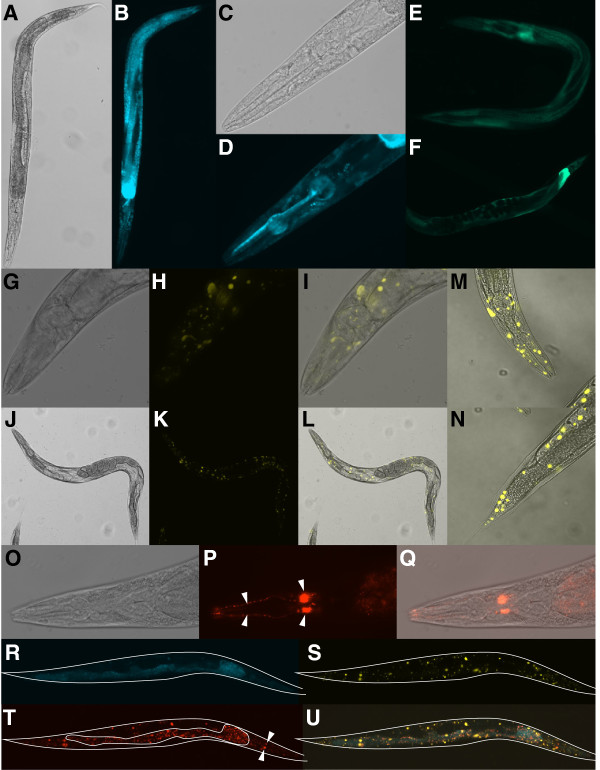
**Expression patterns.** Reporter expression patterns in adult hermaphrodites transgenic for fosmid-based constructs that tag each of the genes *F09E5.3* (**A**-**F**), *F09E5.15* (*prdx*-2) (**G**-**N**) and *EEED8.6* (*ccpp*-6) (**O**-**Q**) individually, or collectively (**R**-**U**). F-CFP (**B**, **D**, **R**), mTFP1 (**E**, **F**), F-YFP (**H**, **I**, **K**, **L**, **M**, **N**, **S**) and Mc-mCherry (**P**, **Q**, **T**) and merged F-CFP/F-YFP/Mc-mCherry (**U**) distributions are presented. DIC was used to observe anatomical details either alone (**A**, **C**, **G**, **J**, **O**) or after merging with the associated FP image(s) (**I**, **L**, **M**, **N**, **Q**). The *C. elegans* strains photographed were CTD1059 (**A**-**D**), CTD1050 (**E, F**), CTD1051 (**G**-**N**), and CTD1052 (**O**-**Q**) and CTD1055 (**R**-**U**) (Additional file 1: Table S7). Images were captured at 100x (**A**, **B**, **E**, **F**, **J**, **K**, **L**, **R**, **S**, **T**, **U**) or 400x (**C**, **D**, **G**, **H**, **I**, **O**, **P**, **Q**) magnification and fluorescence image capture times were 2 msec (**A**, **C**, **G**, **H**, **J**, **K**, **O**, **U**), 5 msec (**S**), 250 msec (**E**, **F**) or 1 sec (**B**, **D**, **P**, **R**, **T**). Confocal images (**M**, **N**) were captured at 400x magnification. Mc-mCherry expression in the anterior dendritic projections and cell bodies of one pair of the four head cephalic neurons are indicated (arrowheads) in panel P. The location of these same cell bodies is also indicated in panel T (arrowheads). In panels R-U the approximate outline of the imaged worm is provided and, in addition, in panel T a region of granular intestinal autofluorescence prominent following the 1 sec excitation of Mc-mCherry is also outlined.

Having generated this pCC1Fos-based resource set we utilized some of its components to build, by iterative rounds of counter-selection recombineering, a series of translational reporter constructs from a single fosmid clone. Although we are using this resource to investigate the transcriptional complexity of operon gene regulation we chose, for the sake of simplicity, to confirm its utility here by tagging genes present on the same fosmid clone but not located within a single operon. A list of fosmid clones each containing three or more such genes with published expression patterns was scanned by eye and clone WRM069dD11, containing three such genes located approximately centrally within the insert, was identified. WRM069dD11 contains the genes *F09E5.3*, *F09E5.15* (*prdx-2*) and *EEED8.6* (*ccpp-6*) that encode, respectively, a putative deoxyribose-phosphate aldolase (DERA), a peroxiredoxin and a metallocarboxypeptidase (Figure 1). The iterative recombineering workflow generated single, double and triple-tagged constructs for both the mTFP1/mCitrine/mCherry and F-CFP/F-YFP/Mc-mCherry FP combinations (Additional file 1: Figure S4 and Figure 2). Transgenic lines were generated for the single- and triple-tagged variants and resulting expression patterns investigated (Figure 3). Microscopic examination of the lines revealed that, as noted previously for the *myo*-3^PROM^-driven transcriptional constructs, the mCitrine and mCherry fusion proteins, even when encoded by CDSs with 2 introns, were, as determined by eye, visibly less bright that the respective F-YFP and Mc-mCherry equivalents. Whatever the cause(s) the low signal levels made discerning clear expression patterns for the two fusion genes, *F09E5.15::mCitrine(2I)* and *EEED8.6::mCherry(2I)*, difficult and further examination of the corresponding lines was not pursued. Interestingly, mTFP1-expressing worms were, consistently, as bright as the otherwise equivalent F-CFP-expressing strains confirming that mTFP1, as encoded by the CDS designed here, is a useful addition to the FP palette for reporter analysis in *C*. *elegans* (Figure 3 panels E, F). All lines transformed with a reporter construct built with one or more of the F-CFP/F-YFP/Mc-mCherry FP set exhibited relatively bright and easily interpretable expression patterns (Figure 3).

Subsequent investigation of expression patterns proceeded by examination of lines transformed with the F-CFP/F-YFP/Mc-mCherry-tagged constructs. *F09E5.3* encodes a DERA homolog that shares 72% similarity and 54% identity with human DERA (data not shown). DERA, as part of the pentose phosphate shunt, catalyzes the reversible reaction of 2-deoxy-D-ribose 5-phosphate to D-glyceraldehyde 3-phosphate and acetaldehyde [31] and, as such, might be expected to exhibit widespread expression consistent with an enzyme involved in intermediate metabolism. Indeed, examination of strains CTD1059 and CTD1055 (Additional file 1: Table S7), that were transformed, respectively, with the *F09E5.3::F-CFP* (fNH058) and the triple gene-tagged construct (fNH086), revealed diffuse F-CFP expression throughout the hypodermis, intestine and pharynx (Figure 3, panels A-D, R). Similarly, strain CTD1050 (Additional file 1: Table S7), transformed with the corresponding *F09E5.3::mTFP1* construct fNH068, exhibited an essentially equivalent expression pattern (Figure 3, panels E, F). This generalized pattern of expression was in agreement with that previously published for strain BC14910 that had been generated by transformation with a corresponding *F09E5.3*^PROM^ transcriptional style reporter construct (Figure 1) [32,33].

*F09E5.15* (*prdx-2*) encodes one of the two 2-Cys peroxiredoxins expressed in *C*. *elegans*. These conserved thioredoxin-coupled peroxidases, *via* their capacity for H_2_O_2_ reduction, are important components of the overall oxidative-stress response employed by multicellular organisms. However, there is also increasing evidence that they have additional, complex functions including important roles as regulators of H_2_O_2_-mediated redox signaling [34]. While the generalized anti-oxidant role might suggest a relatively widespread distribution for PRDX-2 in *C*. *elegans* the previously reported expression patterns indicated a more tissue-, even cell-specific localization suggesting that PRDX-2 may play one of these more discrete, specialized functional role(s). For example, using a *F09E5.15*^PROM^ transcriptional reporter (Figure 1), Isermann et al. (2004) ref. [35] reported that PRDX-2 expression was restricted to the pharyngeal interneurons I2 and I4. A more distributed PRDX-2 expression pattern, including head and tail neurons and intestine, was reported in strain BC13145 transformed with a similar transcriptional reporter (Figure 1) [32,33]. Olahova et al. (2008) ref. [36] demonstrated subsequently, *via* an immunohistochemical approach, that PRDX-2 was also expressed in gonad and intestine. However, examination of strain CTD1051 (Additional file 1: Table S7), transformed with the single *F09E5.15::F-YFP* construct (fNH058), demonstrated a highly unusual expression pattern that did not conform with either of these previous reports (Figure 3, panels G-N). F-YFP expression was observed as intense punctate foci distributed, apparently randomly, throughout the body and that did not co-localize with any specific tissue or cell type. Equivalent patterns were observed in other lines transformed with fNH058 as well as those generated from the triple-tagged construct (Figure 3, panel S). This pattern of concentrated fluorescent foci is difficult to interpret in the context of PRDX-2 function. It might reflect some uncharacterized role but, and possibly most likely, may also result from unexpected, artifactual aggregation of the fusion protein perhaps initiated and promoted by the presence of the *C-*terminal F-YFP sequence. Whilst this is entirely possible, one of us (IAH), with considerable experience in interpreting gene expression patterns in *C*. *elegans*, has not previously observed such aggregations suggesting that if they do occur with reporter gene fusions it is a rare event. We were subsequently made aware (E. Veal, pers. comm.) that very similar punctate expression was observed with a *prdx-2::gfp* translational reporter fusion but not when an N-terminal *gfp::prdx-2* construct was used. Taken together these results indicate that the punctate expression pattern, most likely artifactual aggregation, is due to the C-terminal extension and appears not to be FP-specific.

*EEED8.6* (*ccpp-6*), the third gene to be tagged within fosmid WRM069dD11, and *ccpp-1* each encode one of two *C*. *elegans* cytosolic carboxypeptidases. CCPP-6 has been identified [37] as a deglutamylase that, in conjunction with tubulin tyrosine ligase-like (TTLL) glutamylases, mediate the respective deglutamylation and glutamylation of tubulin thereby regulating the microtubular network present in the *C*. *elegans* neuronal sensory cilia. A *ccpp-1::gfp* transcriptional reporter revealed CCPP-1 expression in head neurons, specifically amphids, which were also demonstrated to exhibit TTLL-dependent tubulin glutamylation activity [37]. Interestingly, a *ccpp-6::gfp* transcriptional reporter suggested labial neuron expression for CCPP-6 whereas, in contrast, a translational version indicated expression in putative amphid cell bodies [37]. This discrepancy was attributed to the inclusion of a possible enhancer(s) in the latter construct. Examination of strain CTD1052 (Additional file 1: Table S7), transformed with the single *EEED8.6::Mc-mCherry* construct (fNH060), demonstrated clear, distinct reporter expression in head neuron cell bodies and anterior dendritic extensions. Careful examination of the relative location of the cell bodies (Figure 3, panel Q) identified these as likely to be the mechanosensitive CEP cephalic neurons. This interpretation was confirmed by additional confocal microscopy (data not shown). Localized CEP neuron expression was observed in other lines transformed with *EEED8.6::Mc-mCherry* (data not shown) and the triple-tagged construct (Figure 3, panel T). Lines transformed with the latter construct exhibited unrelated punctate fluorescence in the red channel due to bleed-through from excitation of the concentrated foci of *F09E5.15::F-YFP*. The clear CEP expression observed here for *EEED8.6* agreed with previously published expression data for strain BC11803 generated by transformation with a corresponding *EEED8.6*^PROM^ transcriptional style reporter (Figure 1) [32,33]. Thus, interestingly, it would appear that CCPP-6 is expressed predominantly in the cells of the cephalic sensilla, whereas CCPP-1, that lacks detectable deglutamylase activity but co-localizes with TTLL-mediated glutamylation [37] is, in contrast, expressed in the amphid neurons.

The use of recombineering to build, directly from a genomic clone, a translational-style *C. elegans* reporter fusion construct is gaining in popularity and a number of related methodologies have been described [5,7-9]. All these different approaches represent variations on a common methodological theme and, although all are welcomed as attempts to make the procedure easier and more robust, they will, as discussed [38], have relative strengths and weaknesses. As is often the case, the choice of which to use will depend upon application and personal preference. We have generated a construct resource that further simplifies the approach used by us enabling the rapid construction of fosmid-based reporter fusion constructs seamlessly tagged, if desired, with multiple FP reporters. From this resource we are making available, through the plasmid depository Addgene, those constructs required to generate fosmid reporters tagged with either mTFP1, F-CFP, F-YFP or Mc-mCherry as these will be of immediate use to others (Tables 1 and 2). Constructs containing sequences encoding mCerulean, mCitrine and mCherry, encoded by the codon-optimized sequences designed by us, are available from CTD.

## Conclusions

We have described the generation and utility of construct collections designed to simplify and facilitate the building of fosmid-based FP reporter constructs *via* counter-selection recombineering. Use of the resource generates final recombineered products that are invariably free of unwanted sequence artefacts and, as such, can be used directly to create transgenic animals with the need for only minimal fidelity checking. Although some of the FPs encoded displayed undesirable characteristics, such as rapid bleaching and poor brightness, the remaining constructs represent a valuable resource that both simplifies the overall procedure and increases significantly recombineering efficiency.

## Methods

### *C. elegans* culture, strains, transformation and microscopy

*C. elegans* culture, handling and manipulations were performed according to standard methods. The wild-type Bristol N2 strain [1] was transformed by co-injection [39] of either plasmid (100 ng/μl) or fosmid (10 ng/μl) DNA together with marker plasmid pRF4 (100 ng/μl). pRF4 contains *rol-6(su1006)* which confers a rolling phenotype by which transformants can be recognized and transgenic lines maintained. Each transgenic strain was established from a different injected animal and therefore is independently generated. FP expression patterns were observed, in hermaphrodites only, by fluorescence microscopy on an Olympus BX61 equipped with DIC optics, filter sets designed to acquire GFP (Chroma, 41012), CFP, mCerulean and mTFP1 (Semrock, CFP-2432A), YFP and mCitrine (Semrock, YFP-2427A) and mCherry (Semrock, mCherry-A) and CellSens (v.1.4) software. Confocal images were captured with a Zeiss LSM510 META system and LSM510 (v.3.4) software.

### General molecular and bioinformatic methods

Unless otherwise stated classical genetic engineering utilized standard protocols [40]. Restriction and modifying enzymes were from NEB (UK) and oligonucleotides (ODNs) from IDT (Belgium). ODNs for priming sequencing or standard PCR reactions were desalted whereas long (>70 nt) ODNs designed to generate linear recombineering amplicons were either PAGE-purified or ultramer grade. All PCRs were performed in volumes of 50 μl containing 15 pmol of each ODN primer and 200 mM of each dNTP and were catalyzed with a high-fidelity DNA polymerase (Phusion, NEB) using conditions designed to minimize mis-incorporation. ODN design, cloning strategies and sequence alignments were performed using MacVector software (MacVector, Inc.). A construct derived directly from a clone originating from the *C. elegans* genomic library constructed in the copy number-inducible fosmid vector pCC1Fos (D. Moerman, pers. comm.) and which thus retains the fosmid backbone is prefixed with an “f” (fosmid). In contrast, non-genomic DNA insert-containing constructs, irrespective of the vector backbone type, are considered plasmids and prefixed “p” (plasmid). Linear RT-cassette-containing counter-selection markers for insertion were PCR-amplified essentially as described [5] using, as template, a restriction fragment excised from the appropriate construct (Additional file 1: Table S6) and an annealing temperature of 50°C.

### Identification of fosmid genomic clones with associated gene expression data

Data from WormBase [41] release WS226 was interrogated to identify candidate pCC1Fos-based genomic clones containing a cluster of genes with associated published expression pattern data. Appropriate filters were applied to these data to remove any data point that did not have an associated reference. The remaining data were then converted into a list of genes by exploiting the high levels of connectivity in the ACeDB [42] schema developed by the WormBase project. The gene co-ordinate data were used to align with the fosmid clone genomic positions by performing a GFF (General Feature Format) union to generate Gene::Fosmid connections. These data were then clustered and sorted on fosmid ID to produce a candidate list of over 4000 clones that contain three or more genes each of which has a published expression pattern.

### Two-step, counter-selection recombineering

All recombineering was undertaken in *E*. *coli* strain MW005 [6] that supports both λ Red- mediated recombineering and copy-number induction of pCC1Fos-based fosmid clones. Positive selection of recombinants *via* the RT-cassette employed tetracycline (Tc, 5 μg/ml) plus chloramphenicol (Cm, 10 μg/ml) to select for the pCC1Fos backbone. On occasion, and as indicated in the text, ampicillin (amp, 50 μg/ml) either replaced, or was used in combination with, Tc. Negative selection – used when selecting for the targeted replacement of the RT-cassette – was achieved with streptomycin (Sm, 500 μg/ml) and Cm (10 μg/ml). Recombineering, when employed at the start of the project as a tool to generate resource components, was performed essentially as described [5]. Subsequent access to these resources enabled the following simplified, more streamlined recombineering protocol to be developed requiring only a single PCR to generate the initial RT-cassette, smaller culture volumes and incubation times, and no formal requirement to sequence final recombinants. A volume (2–3 ml for each RT-cassette-containing PCR product to be introduced) of liquid medium (SOB (−Mg) plus Cm), was inoculated with an aliquot (1 in 10 v/v) from an overnight ‘target fosmid clone’-containing MW005 mini-culture (150 r.p.m., 32°C), incubated on (approx. 2 h, 150 r.p.m., 32°C) to mid log phase (Ab600 approx. 0.6), aliquots (1 ml) transferred into each of an appropriate number of micro-tubes, centrifuged, supernatant removed, cells resuspended (1 ml) in pre-warmed (45°C) medium and the tube(s) transferred to a shaking incubator (Labnet VorTemp, 100 r.p.m., 45°C, 5 min). Each tube was removed, chilled on ice (10 min), harvested by brief centrifugation, cells washed (2 × 1 ml ice-cold ddH_2_O), resuspended (50 μl, ddH_2_O) and electroporated with an appropriate, purified (Promega, Wizard) RT-cassette-containing PCR product (500 ng). Recovery (3 h, 32°C) and subsequent positive selection of colonies harbouring RT-cassette-containing recombinant clones on LB-agar (Tc, Cm, 36-48 h, 32°C) was performed as described [5]. Subsequently, the RT-cassette was replaced, *via* negative-selection recombineering in MW005 cells prepared as described above, with the appropriate gel-purified *Not*I-*Not*I fragment (50 ng) excised from the correct ‘replacement’ construct (see below). Selection of desired recombinant-containing colonies on No Salt (NS)-LB agar (Sm, Cm, 36-48 h, 32°C), subsequent propagation in liquid culture, induction of copy-number (‘CopyControl’, Epicentre) and fosmid DNA isolation were performed as described [5]. Gross fidelity of final constructs was determined by RE analyses.

### Coding sequence design and building pGOv5- and pPD95.86-based sub-clones

DNA sequences encoding the FPs mCerulean [18], mTFP1 [19], mCitrine [20] and mCherry [17], plus *N-* and *C-*TAP-tags, were codon-optimized for expression in *C. elegans* and, by careful placement of appropriate RE sites, designed to be modular in nature to facilitate subsequent sub-clone creation (detailed in supplemental data and Additional file 1: Figure S1). The sequences were commercially synthesized and provided in proprietary vectors. The lack of common RE sites in one of these, pGOv5, facilitated the subsequent generation in this plasmid vector of sets of sub-clones, one for each FP CDS, representing those module combinations likely to be most useful. These included *N-* and *C-*TAP-tagged FPs and FP CDSs containing one or two artificial introns (Additional file 1: Table S1). Following generation of these pGOv5-based sub-clones a number of the insert sequences, together with sequences encoding F-CFP, F-YFP and Mc-mCherry, were each transferred into the backbone of the *myo*-3^PROM^-containing vector pPD95.86 [4] (Additional file 1: Table S1).

### Generating pCC1Fos-based constructs

Both classical genetic engineering and recombineering were employed to, firstly, introduce the RT-cassette into pCC1Fos and, subsequently, build a set of pCC1Fos-based constructs (Additional file 1: Figure S2) designed to simplify the counter-selection recombineering protocol. To retrofit pCC1Fos with the RT-cassette, two separate regions of the pGOv5 backbone were PCR-amplified from pNH002 (Additional file 1: Table S1) DNA (1 ng). One resulting amplicon, generated with ODNs 12015/12016 (Additional file 1: Table S3), comprised approx. 600 bp of the 3^′^ end of the *b*-lactamase (*bla*) coding sequence flanked, at one end, by a stretch (50 bp) identical to a region within pCC1Fos and, at the other, by 50 bp equivalent to one end of the RT-cassette (Additional file 1: Figure S2i). The second amplicon, generated using ODNs 12017/12018 (Additional file 1: Table S3), comprised approx. 200 bp equivalent to a section within pCC1Fos and a 50 bp terminus identical to the other end of the RT-cassette (Additional file 1: Figure S2i). These two amplicons were fused with the RT-cassette in a 3-way Overlap-PCR with ODNs 12015/12018 generating an RT-cassette product with 50 and 200 bp terminal sequences identical to regions in pCC1Fos (Additional file 1: Figure S2ii). The fused product was inserted, *via* positive-selection recombineering, into pCC1Fos, resulting in the replacement of the *LacZ-α* and *loxP* regions and introduction of the partial 3^′^ end of *bla* to generate the RT-cassette-containing pCC1Fos-based construct pNH034 (Additional file 1: Figure S2iii). Subsequently, FP cassettes, excised from the pGOv5-based constructs pNH002, pNH006-8, pNH0013-20 and pNH0026-29 (Additional file 1: Table S1) with *Fsp*I and *Lgu*I (Additional file 1: Figure S2iv), were each recombineered into pNH034 generating a series of constructs in which the RT-cassette had been replaced by a different *Not*I-flanked FP CDS or derivative (Additional file 1: Table S4). Because the full-length *bla* gene was reconstructed during RT-cassette replacement successfully engineered recombinants were selectable with ampicillin (Additional file 1: Figure S2v). Finally, an RT-cassette, PCR-amplified with appropriate ODN pairs (Additional file 1: Tables S3 and S4) (Additional file 1: Figure S2vi) was introduced centrally, *via* positive selection recombineering (Tc, amp), into the FP CDS of each of these constructs generating the corresponding RT-cassette-containing derivatives (Additional file 1: Figure S2vii and Table S4).

A similar protocol was employed to generate an equivalent set of constructs containing F-CFP, F-GFP, F-YFP and Mc-mCherry CDSs. First, an RT-cassette, PCR-amplified with ODNs 12024/12025 (Additional file 1: Table S3) (Additional file 1: Figure S3i), was introduced into the pCC1Fos-based construct pNH043 (Additional file 1: Table S4) *via* positive-selection recombineering (Tc, amp), creating the intermediate pNH038 (note that constructs were not necessarily numbered sequentially) in which the mTFP1 CDS was replaced precisely by the RT-cassette but the flanking *Not*I sites were retained (Additional file 1: Figure S3ii). Next, F-CFP, F-GFP and F-YFP CDSs were PCR-amplified from, respectively, pPD136.61, pPD95.77 and pPD136.64 [4], using ODNs 12028/12029 (Additional file 1: Table S3), and Mc-mCherry from pAA64 [16] using ODNs 12026/12027 (Additional file 1: Table S3) (Additional file 1: Figure S3iii). The resulting PCR products replaced the RT-cassette in pNH038, *via* negative-selection recombineering, generating constructs pNH039-pNH042 (Additional file 1: Table S4) (Additional file 1: Figure S3iv). Finally, an RT-cassette, PCR-amplified using either ODN pairs 12030/12031 or 12032/12033 (Additional file 1: Table S3) and designed to target, respectively, the central region of the F-CFP/F-GFP/F-YFP and Mc-mCherry CDSs, was recombineered, *via* positive-selection recombineering (Tc, amp), into each of pNH039-42 to generate, respectively, pNH050-53 (Additional file 1: Figures S3v & S3vi and Additional file 1: Table S4).

DNA from each of these pCC1Fos-based constructs (Additional file 1: Tables S4) was isolated from appropriate individual ‘mini-cultures’ (5 ml, LB, Cm) in which fosmid copy number had been induced. A *Not*I-*Not*I fragment, encompassing the ‘selection’ or ‘replacement’ cassette, was excised from each, gel-purified, resuspended in water to either 1 or 100 ng/μl, depending on whether the fragment was to be used as, respectively, an RT-cassette PCR template or a direct replacement sequence, and stored in aliquots at −20°C until use.

### Construction of singly and multiply-tagged fosmid-based reporters *via* iterative counter-selection recombineering

Following their generation a subset of these constructs were employed as reagents during iterative rounds of counter-selection recombineering, performed as described above, designed to tag each of the genes *F09E5.3*, *F09E5.15* (*prdx*-2) and *EEED8.6* (*ccpp*-6), located on the same fosmid genomic clone WRM069dD11, with a different FP CDS followed by derivation of double- and triple-tagged reporter constructs. Parallel workflows were initiated – one to incorporate the F-CFP, F-YFP and Mc-mCherry CDSs and two others to insert either the contiguous or two intron-containing sets of CDSs encoding mTFP1/mCitrine/mCherry designed and synthesized as part of the current work (Additional file 1: Figure S4). Briefly, a series of RT-cassettes were PCR-amplified, using ODNs (Additional file 1: Table S5) designed to amplify the cassette from different *Not*I-*Not*I fragment templates isolated from the appropriate pCC1Fos-based selection construct (Additional file 1: Table S5), generating discrete RT-cassettes flanked by approx. 200 bp from the 5^′^ and 3^′^ ends of the final FP CDS to be inserted and with terminal homology arms designed to insert the complete PCR product into the target gene precisely 15 bp (5 codons) upstream of the stop codon (Figure 2). In order that the final fusion gene sequence terminated at the translation stop codon of the native gene the intention was for each reverse recombineering ODN to be designed to anneal immediately 5^′^ to the FP CDS stop signal. Such ODNs were designed successfully to generate RT-cassettes corresponding to the F-CFP, F-YFP and Mc-mCherry CDSs but the presence of *two* in-frame stop codons terminating each of the contiguous and intron-split codon-optimized FP CDSs was overlooked during ODN design resulting in an in-frame TAA triplet being incorporated into each associated RT-cassette (Additional file 1: Table S2) leading to final fusion reporter sequences lacking the last 5 codons of the endogenous gene (Figure 2).

## Competing interests

The authors declare that they have no competing interests.

## Authors’ contributions

NH generated all constructs, with assistance from MW, and transgenic *C*. *elegans* strains with assistance from MSG. NH undertook fluorescence microscopy and expression patterns were interpreted with guidance from IAH. PD provided bioinformatics help and analysis. CTD supervised NH, MW and MSG. NH and CTD wrote the manuscript. All authors read and approved the final manuscript.

## Supplementary Material

Additional file 1 **Hirani et al Additional methods and data file [**[4]**,**[16-20]**,**[43-46]**].** Click here for file
