# Supplementary material for: A simplified counter-selection recombineering protocol for creating fluorescent protein reporter constructs directly from C. elegans fosmid genomic clones
Source: BMC Biotechnol. 2013 Jan 3;13:1. doi: 10.1186/1472-6750-13-1 (PMC3561212; doi:10.1186/1472-6750-13-1)
Supplement: Additional file 1 — Hirani et al Additional methods and data file [[4],[16-20],[43-46]]. [file 1472-6750-13-1-S1.pdf]

Supplementary methods

Design and synthesis of codon-optimized DNA sequences and derivation of pGOv5-based sub-clones

DNA sequences were designed (Gene Designer, DNA 2.0) to be codon-optimized for expression in *C. elegans*, using a codon frequency exclusion threshold of 12%, and to be flanked by unique RE sites to facilitate subsequent manipulation and sub-clone derivation. Designed sequences encoded the FPs mCerulean [18], mTFP1 [19], mCitrine [20] and mCherry [17] and, in addition, an N-TAP-tag and C-TAP-tag designed to fuse, respectively, to the *N*- or *C*-termini of each FP (Fig. S1). By 'fixing' the choice of 2 to 3 adjacent codons, prior to reverse-translation, at least two unique internal blunt-cutting RE sites were included in each FP coding sequence (CDS) to enable facile post-synthesis introduction of artificial introns. mCerulean and mCitrine CDSs included the monomerizing mutation A206K associated with *Aequorea* GFP variants [43]. All FP sequences were, within the constraints of design and codon-optimization criteria, also designed to be, where possible, different from one another in order to minimize the risk of intra-molecular recombination when two or more CDSs would be present in the same, final construct. Both versions of the TAP-tag were designed to be fused to the FP through a flexible [$(G_4S)_3$] linker followed by the internal affinity tag StrepTag II [WSHPQFEK] [44], a short [G_4S] linker, a PreScission protease site [LEVLFQGP] [45], a second [G_4S] linker and, finally, the external affinity tag S-Tag [KETAAAKFERQHMDS] [46] (Fig. S1, panels A & B). For ease of post-synthesis sub-clone derivation the C-TAP-tag was followed, additionally, by a sequence [PKKKRKVGGGPKKKRKV] containing two contiguous SV40 nuclear localization signals (2xNLS) flanked by *Msc*I and *Afe*I sites to enable its subsequent excision. All coding sequences were terminated with two contiguous stop codons (TAA-TAG). Sequences, encoding the N-TAP-tag and C-TAP-tag, the latter fused, in-frame, to mTFP1 (Fig. S1, panel B), were commercially synthesized (Gene Oracle) in the proprietary RE site-depleted vector pGOv5 and designated pNH001 and pNH002, respectively. Sequences encoding mCitrine, mCherry and mCerulean were synthesized (Mr Gene) in the

proprietary vector pMK and the resulting plasmids designated pNH003, pNH004 and pNH005, respectively. All FP sequences lacked the *N*-terminal Met-Val residues and *C*-terminal Lys residue present in their respective native sequences.

A series of sub-clones were built up from the original synthesized units by standard sub-cloning procedures (Table S1). Briefly, each FP-encoding sequence was first excised, as a *Kas*I-*Sac*I fragment, from each of pNH003, pNH004 and pNH005 and introduced into the *Kas*I-*Sac*I-restricted and gel-purified backbone of pNH002 to generate, respectively, pNH006, pNH007 and pNH008 each containing an N-TAP-tag::[$(G_4S)_3$]::FP style CDS. Excision of an *Nae*I-*Sfo*I section from within each of pNH002, pNH006, pNH007 and pNH008 removed the [$(G_4S)_3$] linker generating, following ligation of the vector backbone, pNH009, pNH010, pNH011 and pNH012, respectively. To generate discrete, intact FP CDSs the N-TAP-tag was excised, as an *Nco*I-*Nco*I fragment, from each of pNH009, pNH010, pNH011 and pNH012 which were then rejoined to generate pNH013 (mTFP1), pNH014 (mCitrine), pNH015 (mCherry) and pNH016 (mCerulean), respectively (Table S1). As a result of the sub-cloning strategy each of these FP sequences contains, in place of the *N*-terminal Met-Val residues and *C*-terminal Lys residue present in the native sequences, respective Met-Ala-Ala and Val-Ser-Ala stretches instead.

To generate constructs encoding open-reading frames of design FP::[$(G_4S)_3$]::C-TAP-tag::2xNLS the [$(G_4S)_3$]::C-TAP-tag::2xNLS region of pNH001 was excised as a *Sac*I-*Kpn*I fragment and inserted into the *Sac*I-*Kpn*I-restricted and gel-purified backbones of pNH013, pNH014, pNH015 and pNH016 generating, respectively, pNH017, pNH018, pNH019 and pNH020 (Table S1). Removal of the *C*-terminal 2xNLS-encoding sequence from each of the latter was achieved by excision of the encompassing *Msc*I-*Afe*I fragment and backbone re-ligation to generate, respectively, pNH026, pNH027, pNH028 and pNH029. Constructs of design FP::[$(G_4S)_3$]::2xNLS were created by removal of C-TAP-tag-containing *Nru*I-*Msc*I fragments from each of pNH017, pNH018, pNH019 and pNH020, generating, following vector re-ligation, pNH030, pNH031, pNH032 and pNH033, respectively (Table S1).

For intron insertion eight pairs of 5' phosphorylated (T4 PNK) 36-nt ODNs (Table S2) were annealed, *via* overlapping complementary 3' stretches, and extended (T4 DNA polymerase) to create eight short (51-bp) blunt-ended, artificial intron sequences designated α, β, γ and B to F and equivalent to those present within a set of the commonly used vectors generated previously by Fire et al. (1990). Introns B, α, β, γ were inserted, at the unique *Eco*RV site present in each of the FP CDSs within pNH013, pNH014, pNH015 and pNH016 generating, respectively, pNH078, pNH079, pNH080 and pNH081 (Table S1). To generate constructs with two artificial introns (2I) sequences C and E were introduced into the unique *Bmg*BI sites within pNH078 and pNH080 to give, respectively, pNH082 and pNH084, whereas introns D and F were inserted into the *Bst*Z171 sites within pNH079 and pNH081 generating, respectively, pNH083 and pNH085 (Table 1). The gene finder package Augustus (http://augustus.gobics.de/) was used to predict splicing fidelity of intron-containing FP CDSs.

Building *myo*-3^{PROM}-driven transcriptional reporters

The FP::[$(G_4S)_3$]::C-TAP-tag::2xNLS units from pNH017 (mTFP1), pNH018 (mCitrine), pNH019 (mCherry) and pNH020 (mCerulean) were excised with *Bam*HI and *Kpn*I and ligated into the *Bam*HI-*Kpn*I-restricted and gel-purified backbone of the *myo*-3^{PROM}-containing pPD95.86 (Fire et al, 1990) generating pNH022, pNH023, pNH024 and pNH025, respectively (Table S1). Intronless mTFP1, mCitrine, mCherry and mCerulean CDSs were removed, as *Bam*HI-*Kpn*I fragments, from pNH013, pNH014, pNH015 and pNH016 and sub-cloned into *Bam*HI-*Kpn*I-restricted, gel-purified pPD95.86 to generate, respectively, pNH047, pNH048, pNH035 and pNH049. The corresponding dual-intron-containing FP coding sequences were excised, with *Bam*HI and *Kpn*I, from pNH082 (mTFP1), pNH083 (mCitrine), pNH084 (mCherry) and pNH085 (mCerulean) and transferred into *Bam*HI-*Kpn*I-restricted pPD95.86 generating pNH086, pNH087, pNH088 and pNH089, respectively (Table S1). Finally, F-CFP and F-YFP and Mc-mCherry CDSs were PCR-amplified from, respectively, pPD136.61, pPD136.64 (Fire et al., 1990) and pAA64 (McNally et al, 2006), with

*Bam*HI-containing forward and *Kpn*I-containing reverse primers (data not shown), and cloned into the equivalent RE sites in pPD95.86 generating pNH074, pNH076 and pNH077 (Table S1).

<u>Generation of T7^{PROM}-tagged FP transcription templates, *in vitro* protein production and immunocapture of *in vitro*-expressed FPs</u>

Linear dsDNA transcription templates, each containing a different, contiguous non-intron-containing FP CDS, were generated by PCR using, as template, pNH013 to pNH016 DNA (5 ng) (Table S1). PCRs were primed with ODNs 12036/12037 (Table S3) the latter introducing a T7^{PROM} sequence in the 5' end of each amplicon. As a positive control an equivalent T7^{PROM}-tagged transcription template, containing a contiguous eGFP CDS, was PCR-amplified using pEGFP-N1 (Clontech) DNA (5 ng) as template and primed with ODNs 12038/12039 (Table S3). Proteins were generated *in vitro* with a coupled transcription-translation system (TnT T7 Quick for PCR DNA, Promega), according to the manufacturer's protocol, using, as transcription template, an unpurified aliquot (5 μl) from each completed PCR. To immunocapture *in vitro*-produced FPs an aliquot (5 μl) from the corresponding transcription-translation reaction mix was incubated (16 h, 4°C on a rotary mixer) with Protein-G agarose beads (1 μl, Protein-G agarose, Millipore) in buffer (PBS/0.2 % Triton X-100 (0.5 ml)) and an aliquot (0.25 ml) of the appropriate, undiluted polyclonal antibody (Living Colors full-fength polyclonal antibody (Clontech) for mCerulean, mTFP1 and mCitrine, RFP/DsRed polyclonal antibody (MBL) for mCherry). Beads were pelleted, washed (3 x PBS/0.2 % Triton X-100), resuspended in wash buffer (0.1ml) and either imaged immediately or stored (4°C) as aliquots (5 μl).

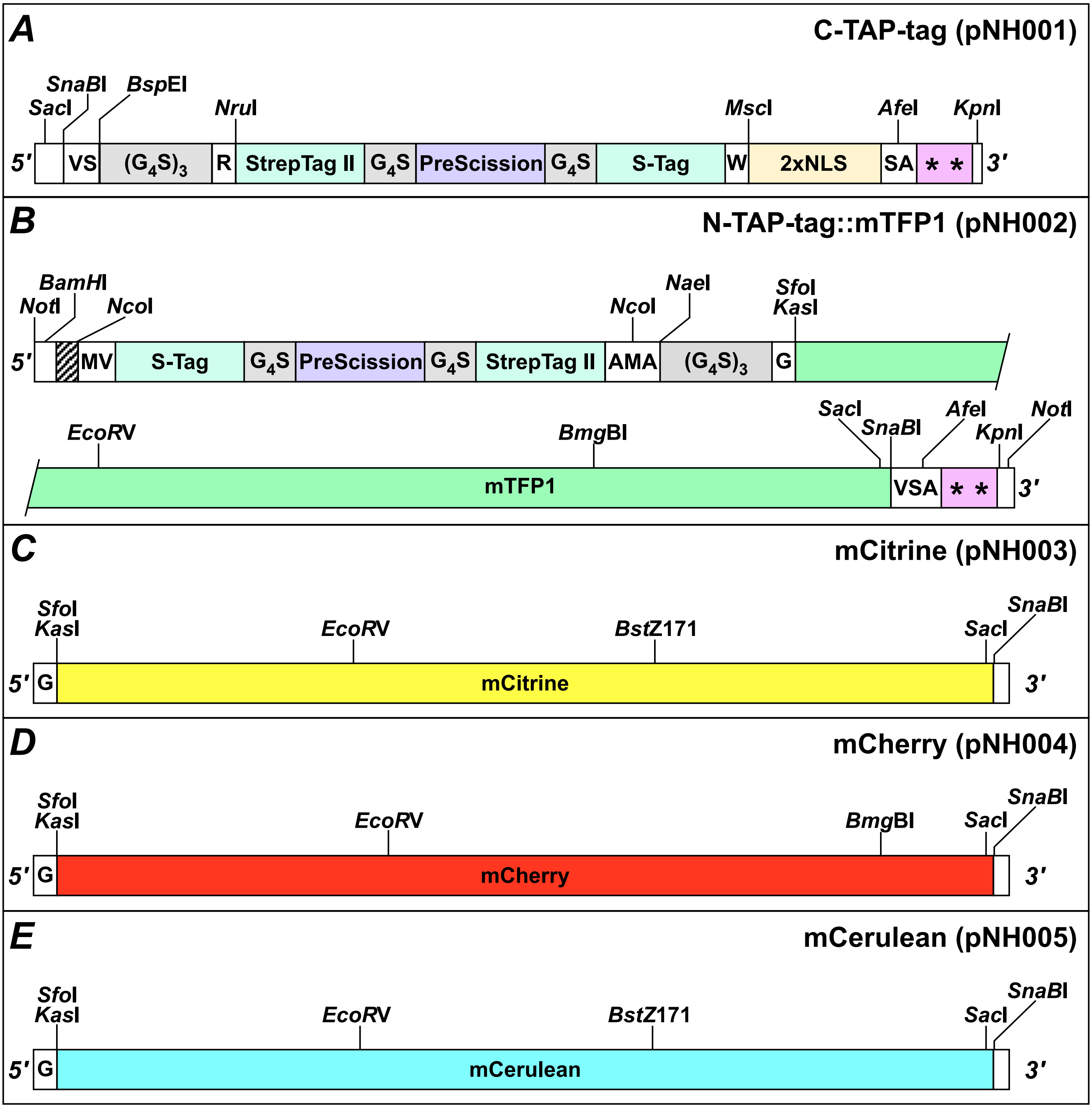

Figure S1. Design and synthesis of fluorescent protein- and TAP-tag-encoding sequences. Sequences encoding a *C*-terminal TAP-tag (Panel A), an *N*-terminal TAP-tag fused, in-frame, to mTFP1 (Panel B), mCitrine (Panel C), mCherry (Panel D) and mCerulean (Panel E) were designed (Gene Designer, DNA 2.0) prior to commercial synthesis. Both TAP-tags were designed with external S-Tag and internal StrepTag II affinity tags flanking a PreScission protease site. A $(G_4S)_3$ linker unit was included in each TAP-tag to separate it from the FP and a pair of G_4S sequences flanked the PreScission site. The *C*-terminal TAP-tag included two in-frame stop codons (TAA-TAG) (asterisks) and a pair of nuclear-localization signals flanked by unique restriction sites. The *N*-TAP-tag included the translation initiation sequence aaaaccATG (hatched box, Panel B). Synthesized *C*-terminal TAP-tag and *N*-terminal TAP-tag::mTFP1 modules resulted in, respectively, plasmids pNH001 (Panel A) and pNH002 (Panel B). mCitrine, mCherry and mCerulean coding sequence units, each containing two unique blunt-cutting restriction enzyme sites to facilitate subsequent artificial intron insertion, were synthesized generating, respectively, plasmids pNH003 (Panel C), pNH004 (Panel D) and pNH005 (Panel E). In all sequences the inclusion of unique restriction enzyme sites, introduced to facilitate downstream sub-cloning and construct manipulation, occasionally required the introduction of one or more additional amino-acid residues absent from the native sequences (open boxes with single letter residues).

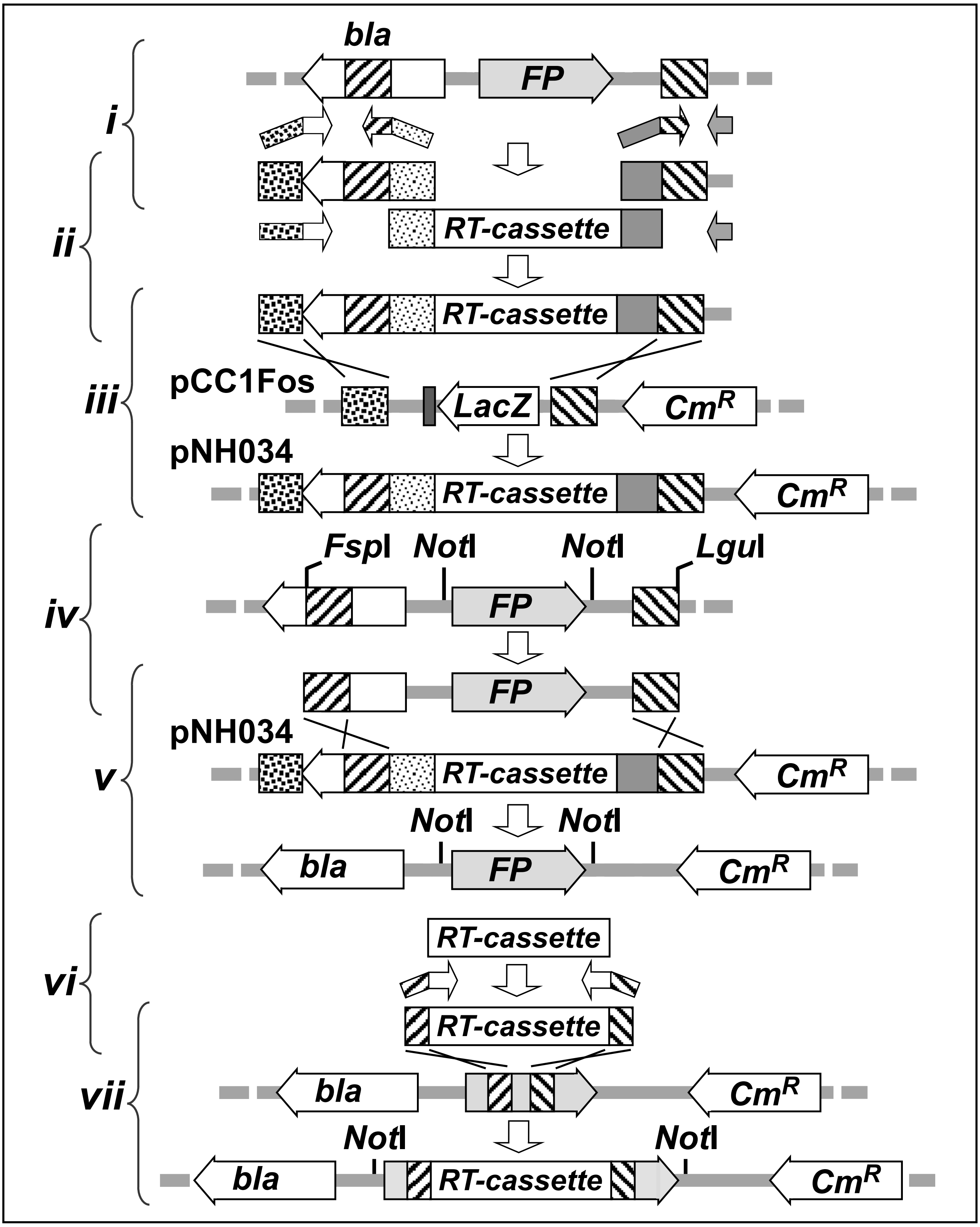

Figure S2. Building a set of pCC1Fos-based recombineering resources based on the codon-optimized FP CDSs designed in the current study. Two regions from the pGOv5 vector backbone were PCR-amplified generating amplicons that overlapped with a RT-cassette-containing sequence (i). The three segments were fused in a 3-way overlap-PCR to generate a single product containing terminal sequences homologous to two discrete regions within the fosmid vector pCC1Fos backbone (ii). This product was introduced into pCC1Fos, via positive selection recombineering, generating the construct pNH034 in which the *Lac*Z-α and loxP sequences have been replaced with the RT-cassette and the 3‘ end of a β-lactamase (*bla*) coding region (iii). Contiguous or artificial intron-containing FP CDSs, excised as *Fsp*I-*Lgu*I fragments from the appropriate pGOv5-based construct (iv), each replaced, via positive selection recombineering, the RT-cassette generating a series of pNH034-derived constructs containing FP CDSs flanked by unique NotI sites enabling subsequent facile excision (v). Finally, a PCR-amplified RT-cassette (vi) was introduced into each of these constructs, *via* positive selection recombineering, replacing the central section of each FP CDS leaving approx. 200 bp 5’ and 3‘ coding sections (vii). Figure not drawn to scale.

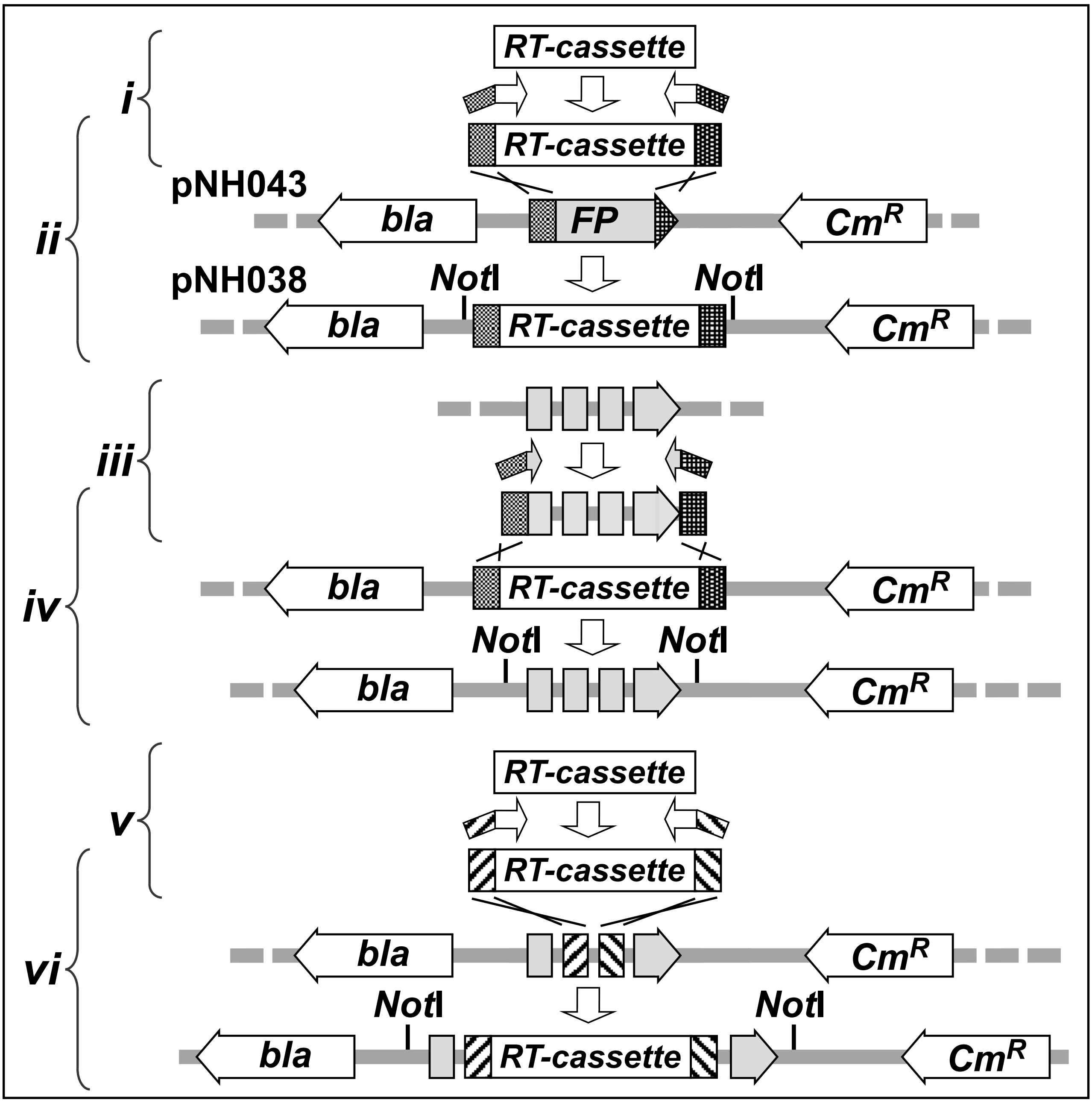

Figure S3. Building a set of pCC1Fos-based recombineering resources for F-CFP, F-GFP, F-YFP and Mc-mCherry FP CDSs. A PCR-amplified RT-cassette (i) was used to replace, *via* positive selection recombineering, the complete mTFP1 CDS present within the pCC1Fos-based construct pNH043 creating pNH038 in which the FP sequence is replaced by the RT-cassette but the useful flanking RE sites are retained (ii). Sequences encoding each of F-CFP, F-GFP, F-YFP and Mc-mCherry were PCR-amplified (iii), from appropriate vectors, and used to replace, via negative selection recombineering, the RT-cassette in pNH038 generating a series of pCC1Fos-based constructs containing NotI-flanked FP CDSs (iv). Finally, a PCR-amplified RT-cassette (v) was introduced, via positive selection recombineering, into each of these replacing the central section of each FP CDS leaving approx. 200 bp 5‘ and 3’ coding sections (vi). Figure not drawn to scale.

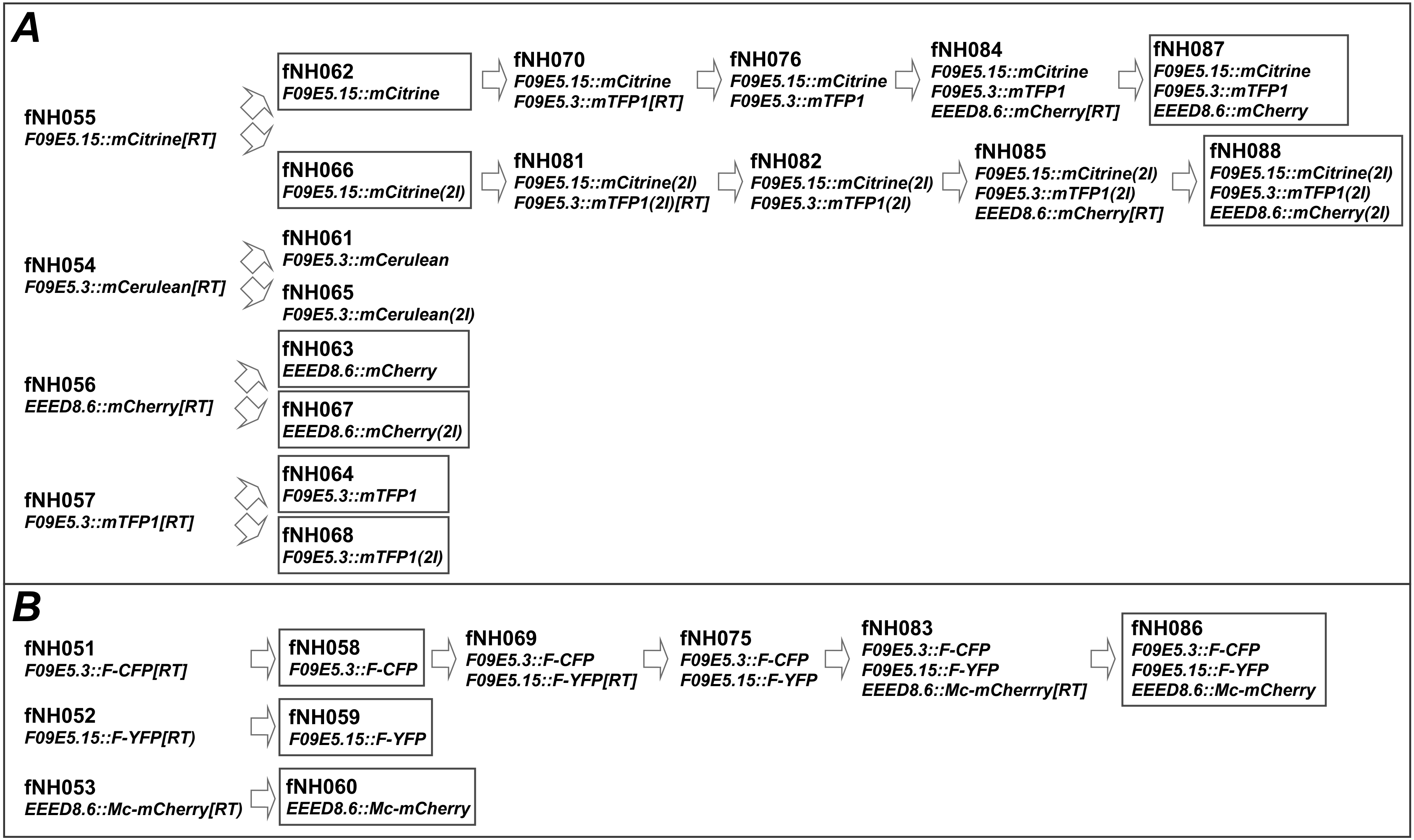

Fig. S4. Fosmid WRM069dD11 counter-selection recombineering workflow. FP CDSs, codon-optimized either as part of this work (Panel A) or by others (Mc-mCherry, [16]) (Panel B) or non-codon-optimized (F-CFP, F-YFP, [4]) (Panel B), were introduced into target genes within the fosmid clone WRM069dD11, *via* iterative rounds of counter-selection recombineering, generating single-, double and triple-tagged reporter constructs. Transgenic lines, created for some of these (boxed), were analyzed for FP reporter expression.

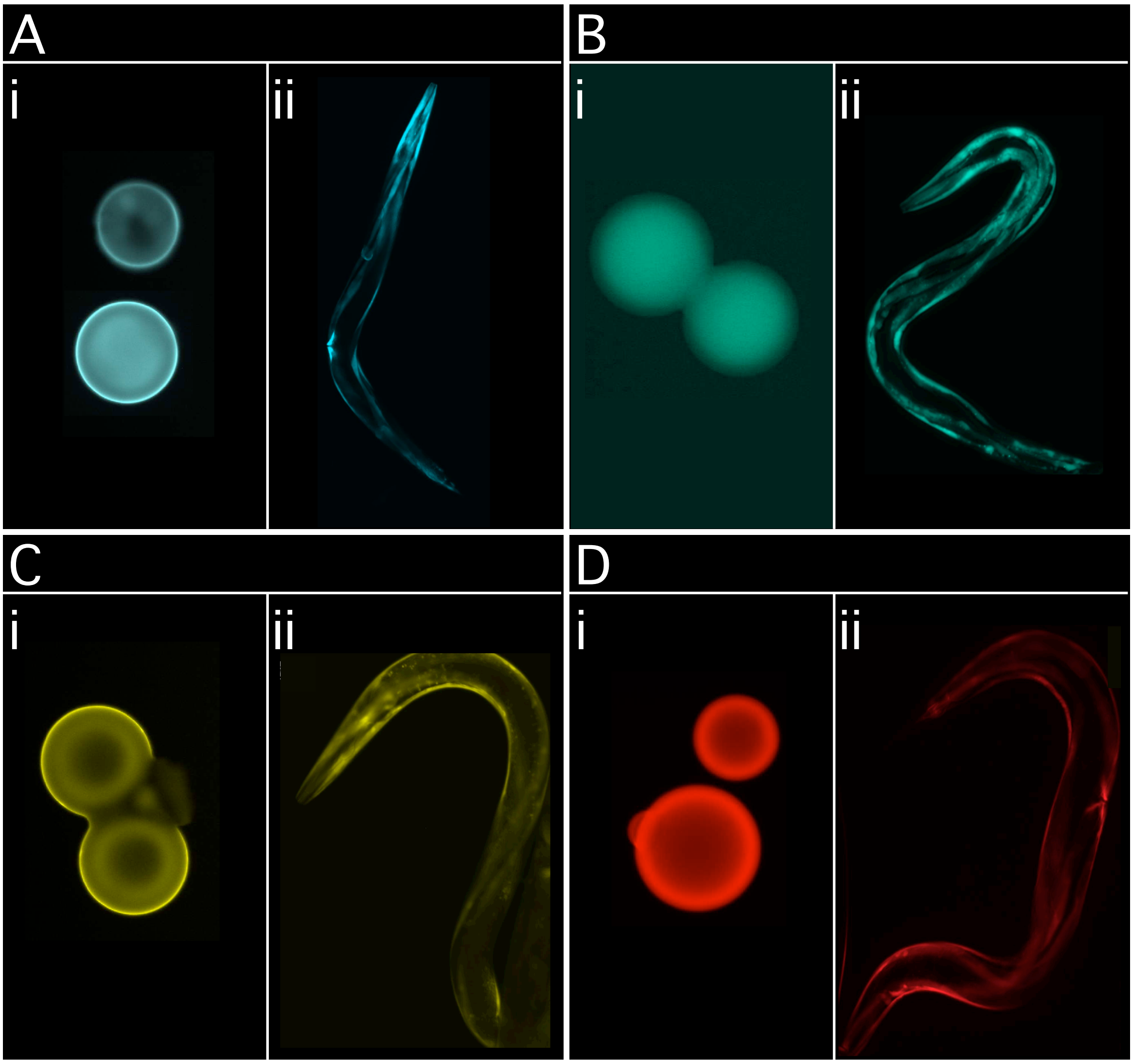

Figure S5. *In vitro* immunocapture and *in vivo* expression of fluorescent proteins encoded by codon-optimized sequences specifically designed for this purpose. Fluorescent proteins mCerulean, mTFP1, mCitrine and mCherry were generated by coupled *in vitro* transcription and translation and immunocaptured to G protein-agarose beads (panels *Ai* - *Di*, respectively). Images of mTFP1-coated beads (e.g. *Bi*) were consistently less sharp than for beads coated with the other fluorescent proteins. *C. elegans* strains, transformed with transgenes from pNH089, pNH086, pNH087 and pNH088 (Table S1) containing, respectively, coding sequences, each with two artificial introns encoding, mCerulean, mTFP1, mCitrine and mCherry (panels *Aii* - *Dii*), were examined for reporter expression. The *myo*-3^{PROM}-driven transgenes directed diffuse reporter expression throughout body muscle cells consistent with the distribution of MYO-3. All images are 200x magnification.

Table S1. pGOv5- and pPD95.86-based sub-clones

Construct	Insert	Vector	Promoter	NLS	Intron 1[d]	Intron 2[d]
pNH001	$[(G_4S)_3]$::C-TAP-tag::2xNLS [a]	pGOv5	-	-	-	-
pNH002	N-TAP-tag::$[(G_4S)_3]$::mTFP1 [b]	pGOv5	-	-	-	-
pNH003	mCitrine [a, b]	pGOv5	-	-	-	-
pNH004	mCherry [a, b]	pGOv5	-	-	-	-
pNH005	mCerulean [a, b]	pGOv5	-	-	-	-
pNH006	N-TAP-tag::$[(G_4S)_3]$::mCitrine	pGOv5	-	-	-	-
pNH007	N-TAP-tag::$[(G_4S)_3]$::mCherry	pGOv5	-	-	-	-
pNH008	N-TAP-tag::$[(G_4S)_3]$::mCerulean	pGOv5	-	-	-	-
pNH009	N-TAP-tag::mTFP1	pGOv5	-	-	-	-
pNH010	N-TAP-tag::mCitrine	pGOv5	-	-	-	-
pNH011	N-TAP-tag::mCherry	pGOv5	-	-	-	-
pNH012	N-TAP-tag::mCerulean	pGOv5	-	-	-	-
pNH013	mTFP1 [c]	pGOv5	-	-	-	-
pNH014	mCitrine [c]	pGOv5	-	-	-	-
pNH015	mCherry [c]	pGOv5	-	-	-	-
pNH016	mCerulean [c]	pGOv5	-	-	-	-
pNH017	mTFP1::$[(G_4S)_3]$::C-TAP-tag::2xNLS	pGOv5	-	Y	-	-
pNH018	mCitrine::$[(G_4S)_3]$::C-TAP-tag::2xNLS	pGOv5	-	Y	-	-
pNH019	mCherry::$[(G_4S)_3]$::C-TAP-tag::2xNLS	pGOv5	-	Y	-	-
pNH020	mCerulean::$[(G_4S)_3]$::C-TAP-tag::2xNLS	pGOv5	-	Y	-	-
pNH022	mTFP1::$[(G_4S)_3]$::C-TAP-tag::2xNLS	pPD95.86	*myo*-3	Y	-	-
pNH023	mCitrine::$[(G_4S)_3]$::C-TAP-tag::2xNLS	pPD95.86	*myo*-3	Y	-	-
pNH024	mCherry::$[(G_4S)_3]$::C-TAP-tag::2xNLS	pPD95.86	*myo*-3	Y	-	-
pNH025	mCerulean::$[(G_4S)_3]$::C-TAP-tag::2xNLS	pPD95.86	*myo*-3	Y	-	-
pNH026	mTFP1::$[(G_4S)_3]$::C-TAP-tag	pGOv5	-	-	-	-
pNH027	mCitrine::$[(G_4S)_3]$::C-TAP-tag	pGOv5	-	-	-	-
pNH028	mCherry::$[(G_4S)_3]$::C-TAP-tag	pGOv5	-	-	-	-
pNH029	mCerulean::$[(G_4S)_3]$::C-TAP-tag	pGOv5	-	-	-	-
pNH030	mTFP1::$[(G_4S)_3]$::2xNLS	pGOv5	-	Y	-	-
pNH031	mCitrine::$[(G_4S)_3]$::2xNLS	pGOv5	-	Y	-	-
pNH032	mCherry::$[(G_4S)_3]$::2xNLS	pGOv5	-	Y	-	-
pNH033	mCerulean::$[(G_4S)_3]$::2xNLS	pGOv5	-	Y	-	-
pNH035	mCherry [c]	pPD95.86	*myo*-3	-	-	-
pNH047	mTFP1 [c]	pPD95.86	*myo*-3	-	-	-
pNH048	mCitrine [c]	pPD95.86	*myo*-3	-	-	-
pNH049	mCerulean [c]	pPD95.86	*myo*-3	-	-	-
pNH074	F-CFP [e]	pPD95.86	*myo*-3	-	n/a	n/a
pNH075	F-GFP [e]	pPD95.86	*myo*-3	-	n/a	n/a
pNH076	F-YFP [e]	pPD95.86	*myo*-3	-	n/a	n/a
pNH077	Mc-mCherry [f]	pPD95.86	*myo*-3	-	n/a	n/a
pNH078	mTFP1[1I] [c]	pGOv5	-	-	B	-
pNH079	mCitrine[1I] [c]	pGOv5	-	-	α	-
pNH080	mCherry[1I] [c]	pGOv5	-	-	β	-
pNH081	mCerulean[1I] [c]	pGOv5	-	-	γ	-
pNH082	mTFP1[2I] [c]	pGOv5	-	-	B	C
pNH083	mCitrine[2I] [c]	pGOv5	-	-	α	D
pNH084	mCherry[2I] [c]	pGOv5	-	-	β	E
pNH085	mCerulean[2I] [c]	pGOv5	-	-	γ	F
pNH086	mTFP1[2I] [c]	pPD95.86	*myo*-3	-	B	C
pNH087	mCitrine[2I] [c]	pPD95.86	*myo*-3	-	α	D
pNH088	mCherry[2I] [c]	pPD95.86	*myo*-3	-	β	E
pNH089	mCerulean[2I] [c]	pPD95.86	*myo*-3	-	γ	F

[a] Lacks intact open-reading frame; [b] FP sequence lacks N-terminal Met-Val and C-terminal Lys residues present in native sequence; [c] N-terminal Met-Val residues and C-terminal Lys residue, present in native coding sequence, replaced, respectively, with Met-Ala-Ala and Val-Ser-Ala; [d] See Table S1 for intron sequences; [e] contains 3 introns [4]; [f] contains 3 introns [16]; n/a – not applicable

Table S2. Artificial intron sequences [a]

ODNs	Sequence (5′-3′)	Intron	Sequence (5′-3′)
4482	GTAAGTTTAAACAGATCCATACTAACTAACTTGTTC		
4483	CTGAAAATTATGTCAGAACAAGTTAGTTAGTATGGA	B	GTAAGTTTAAACAGATCCATACTAACTAACTTGTTCTGACATAATTTTCAG
4484	GTAAGTTTAAACATATATATACTAACTAACCCTGAT		
4485	CTGAAAATTTAAATAATCAGGGTTAGTTAGTATATA	α	GTAAGTTTAAACATATATATACTAACTAACCCTGATTATTTAAATTTTCAG
4486	GTAAGTTTAAACAGTTCGGTACTAACTAACCATACA		
4487	CTGAAAATTTAAATATGTATGGTTAGTTAGTACCGA	β	GTAAGTTTAAACAGTTCGGTACTAACTAACCATACATATTTAAATTTTCAG
4488	GTAAGTTTAAACATGATTTTACTAACTAACTAATCT		
4489	CTGAAAATTTAAATCAGATTAGTTAGTTAGTAAAAT	γ	GTAAGTTTAAACATGATTTTACTAACTAACTAATCTGATTTAAATTTTCAG
4490	GTAAGTTTAATTAAGTTGATACTAACTAACAAAGAT		
4491	CTGAAAATTAATCAGATCTTTGTTAGTTAGTATCAA	C	GTAAGTTTAATTAAGTTGATACTAACTAACAAAGATCTGATTAATTTTCAG
4492	GTAAGTTTAAACAGGATCTTACTAACTAACATGCTA		
4493	CTGAAAATTCAGTGTTAGCATGTTAGTTAGTAAGAT	D	GTAAGTTTAAACAGGATCTTACTAACTAACATGCTAACACTGAATTTTCAG
4494	GTAAGTTTAAACTATTCGTTACTAACTAACTTTAAA		
4495	CTGAAAATTTAAATGTTTAAAGTTAGTTAGTAACGA	E	GTAAGTTTAAACTATTCGTTACTAACTAACTTTAAACATTTAAATTTTCAG
4496	GTAAGTTTAAACAGTTGAATACTAACTAACGGAGAT		
4497	CTGAAAATTTCAAAGATCTCCGTTAGTTAGTATTCA	F	GTAAGTTTAAACAGTTGAATACTAACTAACGGAGATCTTTGAAATTTTCAG

[a] For each pair of ODNs the corresponding intron formed following annealing and extension is indicated alongside.

Table S3. Oligonucleotide sequences

No	sequence (5′-3′)[a]
12015	ccgggctgcatccgatgcaagtgtgtcgctgtcgacgagctcgcgagctcGTCTGACGCTCAGTGGAACGAAAACTCAC
12016	gtgaacaccgtcatatctcgacagcgaccatggAGTGCTGCCATAACCATGAGTGATAAC
12017	ccaattctcatgtttgacagcttatcatcccatggGCTGAGGGGCTGGCCGCTCACAATTC
12018	CGCGTTGGCCGATTCATTAATGCAGC
12024	accgctacacttgccagcccctcagcgcggccgctagcggatccttaaaaGCTGTCGAGATATGACGGTGTTCA
12025	atgttgtgtggaattgtgagcggccagcccctcagcgcggccgcggtaccTCTTGGAGTGGTGAATCCGTTAGC
12026	accgctacacttgccagcccctcagcgcggccgctagcggatccttaaaaATGGTCTCAAAGGGTGAAGAAGATA
12027	atgttgtgtggaattgtgagcggccagcccctcagcgcggccgcggtaccTTAGGATCCACTAGTCTTATACAAT
12028	accgctacacttgccagcccctcagcgcggccgctagcggatccttaaaaATGAGTAAAGGAGAAGAACTTTTCA
12029	atgttgtgtggaattgtgagcggccagcccctcagcgcggccgcggtaccCTATTTGTATAGTTCATCCATGCCA
12030	tactaactaaccctgattatttaaattttcagccaacacttgtcactactGCTGTCGAGATATGACGGTGTTCA
12031	acgcttccatcttcaatgttgtgtctaattttgaagttctgaaaatttaaTCTTGGAGTGGTGAATCCGTTAGC
12032	attatttaaattttcaggtaactaaaggcggaccattaccattcgcctggGCTGTCGAGATATGACGGTGTTCA
12033	gattagttagttagtaaaatcatgtttaaacttacctcagcatcgtaatgTCTTGGAGTGGTGAATCCGTTAGC
12036	GTATGTTGTGTGGAATTGTGAGCGG
12037	GCAGCTAATACGACTCACTATAGGGTGTGGTGGTTACGCGCAGCGTGA
12038	GCAGCTAATACGACTCACTATAGGTTACCGGCCGCCACCATGGTGAGCAAGGGCGAGGAGCTGT
12039	CTCTACAAATGTGGTATGGCTGATT
12042	tactacagccttcgcatatggaaatcgagcattcacaaaatacccagacgGCTGTCGAGATATGACGGTGTTCA
12043	ccaacaacagtttatgcttcacgtcacctttcaaaactccatctctgacaTCTTGGAGTGGTGAATCCGTTAGC
12044	atatggcttgatgtgtttcgcaagatatccagatcacatgaagcaacacgGCTGTCGAGATATGACGGTGTTCA
12045	cttccatcctcgatgttgtgacggattttgaaattaactttaattccgttTCTTGGAGTGGTGAATCCGTTAGC
12046	gtctccacagtttatgtacggttcgaaggcatatgttaagcatccggcggGCTGTCGAGATATGACGGTGTTCA
12047	tgagcttaagtcgttgctttatttctcccttaagagcaccgtcctctgggTCTTGGAGTGGTGAATCCGTTAGC
12048	atggggtgtccaatgctttgctagatatccagaccatatgaaacagcatgGCTGTCGAGATATGACGGTGTTCA
12049	gaaccgtcttcaatattatgtctaatcttaaagttggccttgataccattTCTTGGAGTGGTGAATCCGTTAGC
12074	ggctcaatcctcacttgttccgcatcggagctagcagtcttcttgatgatATGAGTAAAGGAGAAGAACTTTTC
12075	aacaaaaaaaaaagatgataacaaaaactgatctaaagacccttcagacaTTTGTATAGTTCATCCATGCCATG
12076	ggactccaggatccgacaccatcaagccaggagtcaaagaaagccaagagATGAGTAAAGGAGAAGAACTTTTC
12077	gggatacgggggaaattagagatgtaagacatttagtgcttcttgaagtaTTTGTATAGTTCATCCATGCCATG
12078	ccgcccgcgcccagaaaactttgaaaaagcaagcgaggaaggtgattcagATGGTCTCAAAGGGTGAAGAAGAT
12079	tggtctttatcagttctaatgtgggcgccaattcatttcatcatcacagtGGATCCACTAGTCTTATACAATTC
12082	ggactccaggatccgacaccatcaagccaggagtcaaagaaagccaagagATGGCCGCCAGTAAGGGTGAGGAG
12083	gggatacgggggaaattagagatgtaagacatttagtgcttcttgaagtaTTAAGCGCTTACGTAGAGCTCATC
12084	ccgcccgcgcccagaaaactttgaaaaagcaagcgaggaaggtgattcagATGGCCGCCAGTAAGGGTGAGGAG
12085	tggtctttatcagttctaatgtgggcgccaattcatttcatcatcacagtTTAAGCGCTTACGTAGAGCTCATC
12086	ggctcaatcctcacttgttccgcatcggagctagcagtcttcttgatgatATGGCCGCCTCAAAAGGAGAAGAA
12087	aacaaaaaaaaaagatgataacaaaaactgatctaaagacccttcagacaTTAAGCGCTTACGTAGAGCTCGTC

[a]For ODNs used in recombineering procedures the homology arm and template annealing sequences are given in lower and upper case, respectively. Premature stop codons in the reverse ODNs 12083, 12085 and 12087 are underlined.

Table S4. Generation of pCC1Fos-based constructs

Construct [a]	Insert sequence [b]	Source [c]	RT-cassette [d]	ODNs [e]
pNH043	mTFP1	pNH013	pNH054	12042,12043
pNH044	mCitrine	pNH014	pNH055	12044,12045
pNH045	mCherry	pNH015	pNH056	12046,12047
pNH046	mCerulean	pNH016	pNH057	12048,12049
pNH058	N-TAP-tag::mTFP1	pNH002	pNH066	12042,12043
pNH059	N-TAP-tag::mCitrine	pNH006	pNH067	12044,12045
pNH060	N-TAP-tag::mCherry	pNH007	pNH068	12046,12047
pNH061	N-TAP-tag::mCerulean	pNH008	pNH069	12048,12049
pNH062	mTFP1::C-TAP-tag::2xNLS	pNH017	pNH070	12042,12043
pNH063	mCitrine::C-TAP-tag::2xNLS	pNH018	pNH071	12044,12045
pNH064	mCherry::C-TAP-tag::2xNLS	pNH019	pNH072	12046,12047
pNH065	mCerulean::C-TAP-tag::2xNLS	pNH020	pNH073	12048,12049
pNH090	mTFP1::[$(G_4S)_3$]::C-TAP-tag	pNH026	pNH094	12042,12043
pNH091	mCitrine::[$(G_4S)_3$]::C-TAP-tag	pNH027	pNH095	12044,12045
pNH092	mCherry::[$(G_4S)_3$]::C-TAP-tag	pNH028	pNH096	12046,12047
pNH093	mCerulean::[$(G_4S)_3$]::C-TAP-tag	pNH029	pNH097	12048,12049
pNH039	F-CFP	12028/12029	pNH050	12030/12031
pNH040	F-GFP	12028/12029	pNH051	12030/12031
pNH041	F-YFP	12028/12029	pNH052	12030/12031
pNH042	Mc-mCherry	12026/12027	pNH053	12032/12033

[a] pCC1Fos-based construct generated; [b] final inserted sequence; [c] source of insert sequence - either a *Fsp*I-*Lgu*I restriction fragment from donor clone (Table S1) or a PCR product generated with the indicated ODN pair (Table S3); [d] Corresponding sub-clone fitted with RT-cassette; [e] RT-cassette generated by PCR-amplification with this ODN pair (Table S3)

Table S5. Fosmid WRM069dD11 counter-selection recombineering: ODNs and RT-cassettes

Target gene[a]	Insertion sequence[b]	ODNs[c] Fwd	Rev	Selection cassette[d]	Replacement cassette[e]
F09E5.3	F-CFP	12074	12075	pNH050	pNH039
F09E5.15 (*prdx*-2)	F-YFP	12076	12077	pNH052	pNH041
EEED8.6 (*ccpp*-6)	Mc-mCherry	12078	12079	pNH053	pNH042
F09E5.3	mTFP1	12086	12087	pNH054	pNH013
F09E5.15 (*prdx*-2)	mCitrine	12082	12083	pNH055	pNH014
EEED8.6 (*ccpp*-6)	mCherry	12084	12085	pNH056	pNH015
F09E5.3	mTFP1(2I)	12086	12087	pNH054	pNH086
F09E5.15 (*prdx*-2)	mCitrine(2I)	12082	12083	pNH055	pNH087
EEED8.6 (*ccpp*-6)	mCherry(2I)	12084	12085	pNH056	pNH088

[a] Gene targets in fosmid genomic clone WRM069dD11; [b] FP CDS to be inserted; [c] ODNs designed to PCR-amplify counter-selection RT-cassette (see Table S3 for sequences); [d] PCR performed using, as template, the RT-cassette-containing *Not*I-*Not*I fragment excised from this construct (also see Table 2); [e] *Not*I-*Not*I fragment containing the FP CDS replacement cassette excised from this construct.

Table S6. pCC1Fos-based resources for counter-selection recombineering

	RT-cassette[b]				Replacement cassette[c]		
Sequence to be inserted[a]	RT	kb[d]	Fwd (5′-3′)[e]	Rev (5′-3′)[e]	pCC1Fos-based	pGOv5-based	kb[d]
RT-cassette	pNH034	2.0	GCTGTCGAGATATGACGGTGTTCA	TCTTGGAGTGGTGAATCCGTTAGC	-	-	-
F-CFP [f]	pNH050	2.4	ATGAGTAAAGGAGAAGAACTTTTC	[*]TTTGTATAGTTCATCCATGCCATG	pNH039	n/a	0.9
F-GFP [f]	pNH051	2.4	ATGAGTAAAGGAGAAGAACTTTTC	[*]TTTGTATAGTTCATCCATGCCATG	pNH040	n/a	0.9
F-YFP [f]	pNH052	2.4	ATGAGTAAAGGAGAAGAACTTTTC	[*]TTTGTATAGTTCATCCATGCCATG	pNH041	n/a	0.9
Mc-mCherry [g]	pNH053	2.4	ATGGTCTCAAAGGGTGAAGAAGAT	[*]GGATCCACTAGTCTTATACAATTC	pNH042	n/a	0.9
mTFP1	pNH054	2.4	ATGGCCGCCTCAAAAGGAGAAGAA	[*]AGCGCTTACGTAGAGCTCGTCCAT	pNH043	pNH013	0.7
mCitrine	pNH055	2.4	ATGGCCGCCAGTAAGGGTGAGGAG	[*]AGCGCTTACGTAGAGCTCATCCAT	pNH044	pNH014	0.7
mCherry	pNH056	2.4	ATGGCCGCCAGTAAGGGTGAGGAG	[*]AGCGCTTACGTAGAGCTCATCCAT	pNH045	pNH015	0.7
mCerulean	pNH057	2.4	ATGGCCGCCTCAAAAGGAGAAGAA	[*]AGCGCTTACGTAGAGCTCGTCCAT	pNH046	pNH016	0.7
N-TAP-tag::[$(G_4S)_3$]::mTFP1	pNH066	2.6	ATGGTTAAAGAAACAGCAGCAGCG	[*]AGCGCTTACGTAGAGCTCGTCCAT	pNH058	pNH002	0.9
N-TAP-tag::[$(G_4S)_3$]::mCitrine	pNH067	2.6	ATGGTTAAAGAAACAGCAGCAGCG	[*]AGCGCTTACGTAGAGCTCATCCAT	pNH059	pNH006	0.9
N-TAP-tag::[$(G_4S)_3$]::mCherry	pNH068	2.6	ATGGTTAAAGAAACAGCAGCAGCG	[*]AGCGCTTACGTAGAGCTCATCCAT	pNH060	pNH007	0.9
N-TAP-tag::[$(G_4S)_3$]::mCerulean	pNH069	2.6	ATGGTTAAAGAAACAGCAGCAGCG	[*]AGCGCTTACGTAGAGCTCGTCCAT	pNH061	pNH008	0.9
mTFP1::[$(G_4S)_3$]::C-TAP-tag::2xNLS	pNH070	2.6	ATGGCCGCCTCAAAAGGAGAAGAA	[*]AGCGCTAACTTTTCGCTTCTTCTT	pNH062	pNH017	0.95
mCitrine::[$(G_4S)_3$]::C-TAP-tag::2xNLS	pNH071	2.6	ATGGCCGCCAGTAAGGGTGAGGAG	[*]AGCGCTAACTTTTCGCTTCTTCTT	pNH063	pNH018	0.95
mCherry::[$(G_4S)_3$]::C-TAP-tag::2xNLS	pNH072	2.6	ATGGCCGCCAGTAAGGGTGAGGAG	[*]AGCGCTAACTTTTCGCTTCTTCTT	pNH064	pNH019	0.95
mCerulean::[$(G_4S)_3$]::C-TAP-tag::2xNLS	pNH073	2.6	ATGGCCGCCTCAAAAGGAGAAGAA	[*]AGCGCTAACTTTTCGCTTCTTCTT	pNH065	pNH020	0.95
mTFP1::[$(G_4S)_3$]::C-TAP-tag	pNH094	2.3	ATGGCCGCCTCAAAAGGAGAAGAA	[*]AGCCCATGAGTCCATATGCTGTCT	pNH090	pNH026	0.9
mCitrine::[$(G_4S)_3$]::C-TAP-tag	pNH095	2.3	ATGGCCGCCAGTAAGGGTGAGGAG	[*]AGCCCATGAGTCCATATGCTGTCT	pNH091	pNH027	0.9
mCherry::[$(G_4S)_3$]::C-TAP-tag	pNH096	2.3	ATGGCCGCCAGTAAGGGTGAGGAG	[*]AGCCCATGAGTCCATATGCTGTCT	pNH092	pNH028	0.9
mCerulean::[$(G_4S)_3$]::C-TAP-tag	pNH097	2.3	ATGGCCGCCTCAAAAGGAGAAGAA	[*]AGCCCATGAGTCCATATGCTGTCT	pNH093	pNH029	0.9
mTFP1::[$(G_4S)_3$]::2xNLS	pNH070	2.6	ATGGCCGCCTCAAAAGGAGAAGAA	[*]AGCGCTAACTTTTCGCTTCTTCTT	n/a	pNH030 [h]	0.8
mCitrine::[$(G_4S)_3$]::2xNLS	pNH071	2.6	ATGGCCGCCAGTAAGGGTGAGGAG	[*]AGCGCTAACTTTTCGCTTCTTCTT	n/a	pNH031 [h]	0.8
mCherry::[$(G_4S)_3$]::2xNLS	pNH072	2.6	ATGGCCGCCAGTAAGGGTGAGGAG	[*]AGCGCTAACTTTTCGCTTCTTCTT	n/a	pNH032 [h]	0.8
mCerulean::[$(G_4S)_3$]::2xNLS	pNH073	2.6	ATGGCCGCCTCAAAAGGAGAAGAA	[*]AGCGCTAACTTTTCGCTTCTTCTT	n/a	pNH033 [h]	0.8
mTFP1[B]	pNH054	2.4	ATGGCCGCCTCAAAAGGAGAAGAA	[*]AGCGCTTACGTAGAGCTCGTCCAT	n/a	pNH078	0.8
mCitrine[α]	pNH055	2.4	ATGGCCGCCAGTAAGGGTGAGGAG	[*]AGCGCTTACGTAGAGCTCATCCAT	n/a	pNH079	0.8
mCherry[β]	pNH056	2.4	ATGGCCGCCAGTAAGGGTGAGGAG	[*]AGCGCTTACGTAGAGCTCATCCAT	n/a	pNH080	0.8
mCerulean[γ]	pNH057	2.4	ATGGCCGCCTCAAAAGGAGAAGAA	[*]AGCGCTTACGTAGAGCTCGTCCAT	n/a	pNH081	0.8
mTFP1[BC]	pNH054	2.4	ATGGCCGCCTCAAAAGGAGAAGAA	[*]AGCGCTTACGTAGAGCTCGTCCAT	n/a	pNH082	0.8
mCitrine[αD]	pNH055	2.4	ATGGCCGCCAGTAAGGGTGAGGAG	[*]AGCGCTTACGTAGAGCTCATCCAT	n/a	pNH083	0.8
mCherry[βE]	pNH056	2.4	ATGGCCGCCAGTAAGGGTGAGGAG	[*]AGCGCTTACGTAGAGCTCATCCAT	n/a	pNH084 [i]	0.8
mCerulean[γF]	pNH057	2.4	ATGGCCGCCTCAAAAGGAGAAGAA	[*]AGCGCTTACGTAGAGCTCGTCCAT	n/a	pNH085	0.8

[a] sequence to be seamlessly inserted into target; [b] the counter-selection RT-cassette should be PCR-amplified from this construct using, as template, the RT-cassette-containing *Not*I-*Not*I fragment; [c] the desired replacement cassette can be excised, as a *Not*I-*Not*I fragment, from either the pCC1Fos- (if available) or parental pGOv5-based (Table 1) constructs; [d] approximate sizes of the isolated *Not*I-fragment (or *Nco*I-*Nco*I fragment for pNH034) to be used as PCR template or replacement sequence; [e] suggested ODN sequences required to PCR-amplify RT-cassette-containing sequence. Reverse ODN sequence lacks a 5′ stop triplet [*]; [f] intron-containing (see ref 4); [g] intron-containing (see ref 16); [h] 3′ homology arm of replacement sequence is 63 bp long; [i] 3′ homology arm of replacement sequence is 100 bp long; n/a = not available

Table S7. Strains generated during the course of the present work

Strain [a]	Genotype [b]	Construct [c]
CTD1025	N2 whEx[*Pmyo-3>mCitrine::C-TAP-TAG::2xNLS*]	pNH023
CTD1026	N2 whEx[*Pmyo-3>mCherry::C-TAP-TAG::2xNLS*]	pNH024
CTD1027	N2 whEx[*Pmyo-3>mCerulean::C-TAP-TAG::2xNLS*]	pNH025
CTD1028	N2 whEx[*Pmyo-3>mTFP1::C-TAP-TAG::2xNLS*]	pNH022
CTD1029	N2 whEx[*Pmyo-3>mCherry*]	pNH035
CTD1030	N2 whEx[*Pmyo-3>mTFP1*]	pNH047
CTD1031	N2 whEx[*Pmyo-3>mCitrine*]	pNH048
CTD1032	N2 whEx[*Pmyo-3>mCerulean*]	pNH049
CTD1033	N2 whEx[*Pmyo-3>F-CFP*]	pNH074
CTD1034	N2 whEx[*Pmyo-3>F-YFP*]	pNH076
CTD1035	N2 whEx[*Pmyo-3>Mc-mCherry*]	pNH077
CTD1036	N2 whEx[*Pmyo-3>mTFP1(2I)*]	pNH086
CTD1037	N2 whEx[*Pmyo-3::mCitrine(2I)*]	pNH087
CTD1038	N2 whEx[*Pmyo-3>mCherry(2I)*]	pNH088
CTD1039	N2 whEx[*Pmyo-3>mCerulean(2I)*]	pNH089
CTD1040	N2 whEx[*F09E5.15::mCitrine(2I)*]	fNH066
CTD1041	N2 whEx[*EEED8.6::mCherry(2I)*]	fNH067
CTD1050	N2 whEx[*F09E5.3::mTFP1(2I)*]	fNH068
CTD1051	N2 whEx[*F09E5.15::F-YFP*]	fNH059
CTD1052	N2 whEx[*EEED8.6::Mc-mCherry*]	fNH060
CTD1053	N2 whEx[*F09E5.3::mTFP1(2I);F09E5.15::mCitrine(2I);EEED8.6::mCherry(2I)*]	fNH088
CTD1054	N2 whEx[*F09E5.15::mCitrine*]	fNH062
CTD1055	N2 whEx[*F09E5.3::F-CFP;F09E5.15::F-YFP;EEED8.6::Mc-mCherry*]	fNH086
CTD1056	N2 whEx[*F09E5.3::mTFP1;F09E5.15::mCitrine;EEED8.6::mCherry*]	fNH087
CTD1057	N2 whEx[*F09E5.3::mTFP1*]	fNH064
CTD1058	N2 whEx[*EEED8.6::mCherry*]	fNH063
CTD1059	N2 whEx[*F09E5.3::F-CFP*]	fNH058

[a] Lab strain name; [b] allele number not shown; [c] see Table 1 and Fig.S1 for plasmid- and fosmid-based construct details
